# Elevated apolipoprotein C3 augments diabetic kidney disease and associated atherosclerosis in type 2 diabetes

**DOI:** 10.1172/jci.insight.177268

**Published:** 2024-05-14

**Authors:** Jocelyn Cervantes, Juraj Koska, Farah Kramer, Shreeram Akilesh, Charles E. Alpers, Adam E. Mullick, Peter Reaven, Jenny E. Kanter

**Affiliations:** 1 Division of Metabolism, Endocrinology, and Nutrition, UW Medicine Diabetes Institute, University of Washington, Seattle, Washington, USA.; 2 VA Phoenix Health Care System, Phoenix, Arizona, USA.; 3 Department of Laboratory Medicine and Pathology, University of Washington, Seattle, Washington, USA.; 4 Ionis Pharmaceuticals, Carlsbad, California, USA.

**Keywords:** Metabolism, Nephrology, Diabetes, Lipoproteins, Macrophages

## Abstract

Diabetes increases the risk of both cardiovascular disease and kidney disease. Notably, most of the excess cardiovascular risk in people with diabetes is in those with kidney disease. Apolipoprotein C3 (APOC3) is a key regulator of plasma triglycerides, and it has recently been suggested to play a role in both type 1 diabetes–accelerated atherosclerosis and kidney disease progression. To investigate if APOC3 plays a role in kidney disease in people with type 2 diabetes, we analyzed plasma levels of APOC3 from the Veterans Affairs Diabetes Trial. Elevated baseline APOC3 levels predicted a greater loss of renal function. To mechanistically test if APOC3 plays a role in diabetic kidney disease and associated atherosclerosis, we treated black and tan, brachyury, WT and leptin-deficient (OB; diabetic) mice, a model of type 2 diabetes, with an antisense oligonucleotide (ASO) to APOC3 or a control ASO, all in the setting of human-like dyslipidemia. Silencing APOC3 prevented diabetes-augmented albuminuria, renal glomerular hypertrophy, monocyte recruitment, and macrophage accumulation, partly driven by reduced ICAM1 expression. Furthermore, reduced levels of APOC3 suppressed atherosclerosis associated with diabetes. This suggests that targeting APOC3 might benefit both diabetes-accelerated atherosclerosis and kidney disease.

## Abstract 2

APOC3 predicts kidney disease in people with type 2 diabetes, and APOC3 depletion prevents diabetic kidney disease by reducing the effect of triglyceride-rich lipoprotein on glomerular cells.

## Graphical abstract

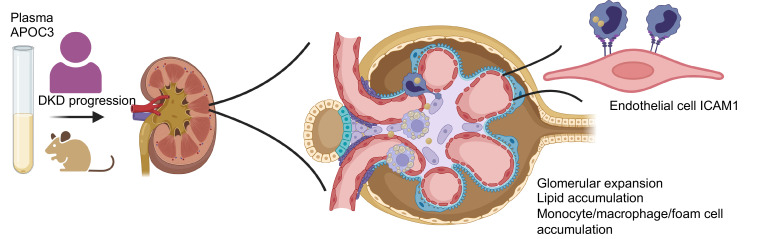

## Introduction

People with diabetes have an increased risk of developing complications, such as diabetic kidney disease and accelerated cardiovascular disease (1). Importantly, impaired kidney function is a strong risk factor for cardiovascular disease (2). Reduced renal function is associated with elevated levels of apolipoprotein C3 (APOC3) (3, 4), a key inhibitor of lipolysis and hepatic clearance of triglyceride-rich lipoproteins (TRLs) (5, 6). In people with type 1 diabetes, plasma APOC3 levels correlate with levels of albuminuria (7, 8), potentially suggesting a role for APOC3 in diabetic kidney disease (DKD). Recently, a link between APOC3 and DKD was demonstrated in people with type 1 diabetes participating in the FinnDiane study. Plasma levels of APOC3 predicted the progression of DKD, defined as the progression of albuminuria or the initiation of renal replacement therapy (8). Furthermore, we, and others, have recently demonstrated that APOC3 levels are also predictors of cardiovascular events in people with type 1 diabetes (8–10).

In humans and mice with human-like dyslipidemia, APOC3 is distributed across most lipoprotein classes, including VLDL, LDL, and HDL particles. HDL-associated APOC3 was not a significant risk predictor of cardiovascular disease in people with type 1 diabetes (9), consistent with the mechanistic mouse models, where VLDL and remnants are the primary pro-atherogenic particles carrying APOC3 (11). Lipids have been shown to accumulate in the kidney in the setting of human diabetic nephropathy (12). Alleviating the stress induced by excess lipid accumulation reduces the progression of DKD in mouse models (13). Exact lipid types that may accelerate DKD in humans are unknown; however, lowering plasma triglycerides using fenofibrates, but not statins, which primarily reduce LDL (14), has been shown to have a small but statistically significant inhibitory effect on the progression of albuminuria in people with diabetes (15–17), potentially suggesting that it is the TRLs that may contribute to DKD.

Together, this implies that APOC3 and TRLs may be driving DKD. To this end, we tested the role of APOC3 in DKD in type 2 diabetes. We show that plasma levels of APOC3 predict the progression of DKD in people with type 2 diabetes and, using mechanistic mouse models that have elevated levels of TRLs, demonstrate that silencing APOC3 reduces atherosclerosis and kidney disease via reduced accumulation of TRLs in the glomerulus and artery wall. Collectively, our observations highlight the possibility that targeting APOC3 may be beneficial for treating atherosclerosis and kidney disease in people with diabetes.

## Results

### Plasma APOC3 predicts DKD progression in humans with type 2 diabetes.

To begin to test the hypothesis that plasma APOC3 plays a role in DKD, APOC3 levels were assessed in baseline plasma samples from the Veterans Affairs Diabetes Trial (VADT) (18, 19). The present study included 1,494 VADT participants with available data on renal function loss (RFL), defined as a 40% or greater decline in estimated glomerular filtration rate (eGFR) at 2 subsequent visits. RFL developed in 198 participants over a median follow-up of 3.5 years (controls were followed for a median of 5.3 years), corresponding to 13.3% of the participants with data on RFL. Baseline characteristics stratified by RFL are outlined in Table 1. Participants who developed RFL, on average, had worse glycemic control, longer history of diabetes, higher systolic blood pressure, higher prevalence of micro- and macroalbuminuria, increased triglycerides and total cholesterol, and lower HDL-cholesterol. In line with the lipid profile, APOC3 levels were elevated in baseline samples in participants who subsequently developed RFL.

**Figure 1:**
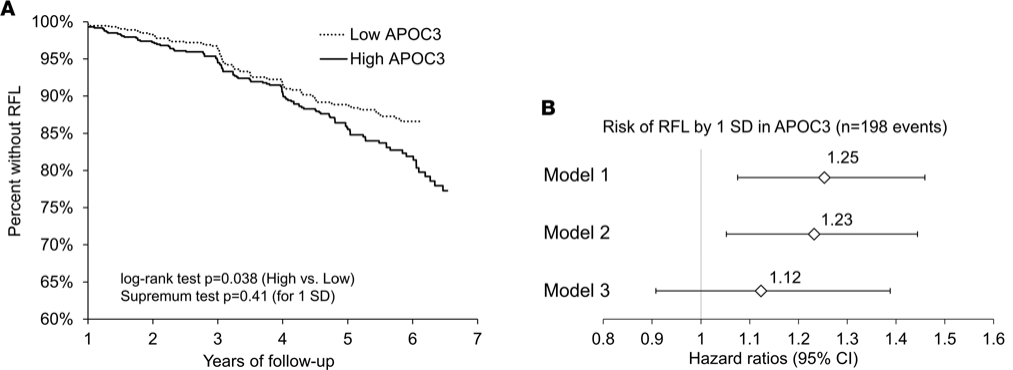
The association between baseline APOC3 and risk of RFL in VADT. (**A**) Kaplan-Meier curves for high and low strata (defined by median, i.e., 11.6 mg/dL) of baseline plasma APOC3. Numbers at risk are as follows (low/high APOC3): year 1: 741/744, year 2: 695/699, year 3: 648/653, year 4: 587/587, year 5: 467/464, and year 6: 198/241. (**B**) Cox proportional hazard regression models for incident RFL in all participants and by the glucose-lowering group. Models were adjusted for glucose-lowering group assignment (model 1) and then adjusted for age, sex, race and ethnicity, diabetes duration, hemoglobin A1c, history of hypertension, systolic blood pressure, albuminuria category, and eGFR at baseline (model 2), and then for model 2 variables and baseline triglycerides (model 3).

Consistent with the idea that APOC3 might play a role in DKD, higher baseline APOC3 levels were associated with an increased risk of RFL in the survival analysis (Figure 1A). After adjustment for treatment allocation (standard or intense glucose control), higher baseline APOC3 was associated with increased RFL risk (hazard ratio per 1 SD in APOC3 1.25 [95% CI 1.08–1.46], *P* = 0.0039, model 1), and the association remained significant after further adjustment for nonlipid clinical and demographic covariates (1.23 [1.05–1.44], *P* = 0.0098, model 2) (Figure 1B). The predictive effect of APOC3 was diminished after adjusting for plasma triglycerides (1.12 [0.91–1.39], *P* = 0.29, model 3, Figure 1B), highlighting the close relationship between APOC3 and plasma triglycerides. The association between APOC3 and RFL was unchanged after adjusting for plasma LDL- or HDL-cholesterol (1.24 [1.05–1.48], *P* = 0.013 and 1.22 [1.04–1.43, *P* = 0.015, respectively).

### APOB-containing lipoproteins accumulate in glomeruli in diabetic nephropathy.

**Figure 2:**
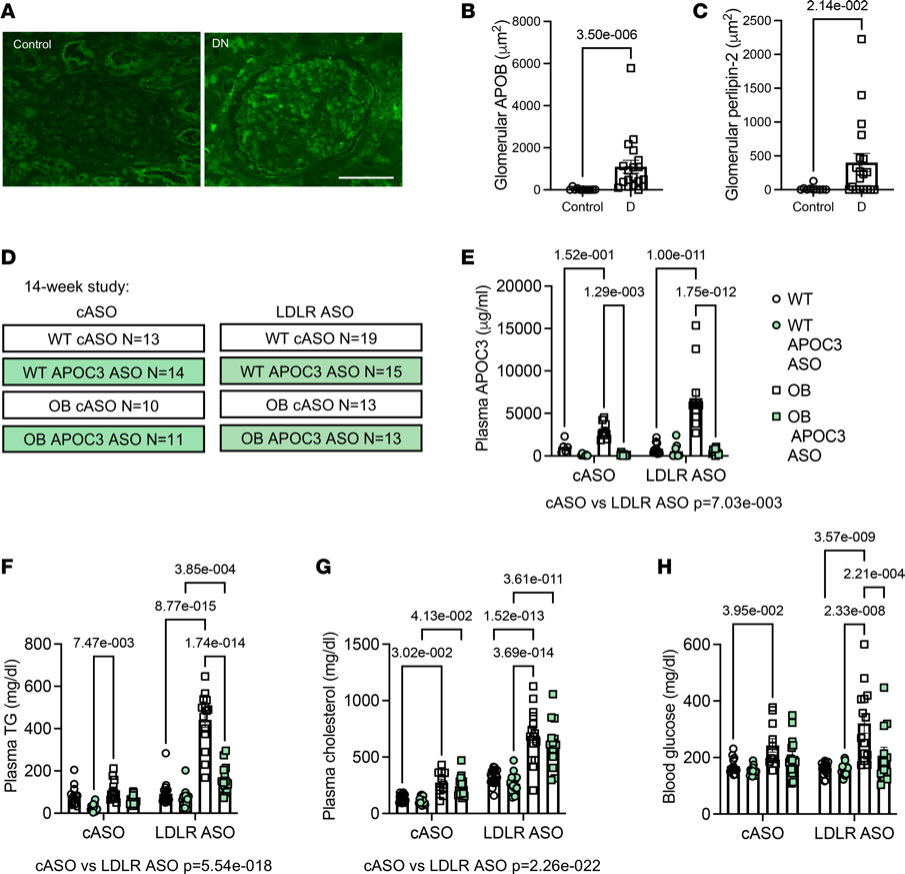
APOB-containing lipids accumulate in the glomerulus in the setting of diabetic nephropathy. (**A**) Representative image of APOB-stained kidney sections from histopathologically healthy controls (*N* = 11) and pathology-verified diabetic nephropathy (*N* = 19). (**B**) Quantification of glomerular APOB staining. (**C**) Quantification of glomerular perilipin 2 staining. (**D**) Schematic of the study plan (14-week study). Briefly, WT and leptin-deficient OB mice were treated with either a control antisense oligonucleotide (cASO) or LDLR ASO. Within each group, a subset was treated with either a cASO or an ASO to APOC3. Mice were then placed on a high-fat diet for 14 weeks. (**E**) Plasma APOC3 levels at 14 weeks (*N* = 5, 7, 8, 11, 8, 11, 11). (**F**) Plasma TG at 14 weeks. (**G**) Plasma cholesterol at 14 weeks. (**H**) Blood glucose at 14 weeks (ad lib–fed morning glucose). Data expressed as mean ± SEM. Data were analyzed by 2-way ANOVA followed by Tukey’s multiple comparisons test. The text under the graph indicates the overall significance between cASO and LDLR ASO groups. *N* as indicated in **D**, unless otherwise noted. For data on other time points, see Supplemental Table 3.

Diabetic nephropathy is associated with increased glomerular lipid accumulation (12). For assessment of whether non-HDL-associated lipids were accumulating in the kidney in the setting of DKD, renal biopsy specimens were obtained and stained for APOB and the intracellular lipid droplet protein perilipin 2. Sections from pathology-verified diabetic nephropathy were compared with histologically healthy kidney sections (Supplemental Table 1; supplemental material available online with this article; https://doi.org/10.1172/jci.insight.177268DS1). The diabetic nephropathy sections ranged from stage 1 to 4. Compared with glomeruli from the nondiabetic controls, glomeruli from people with diabetic nephropathy were larger (glomerular area: 12,759 ± 2,058 μm^2^ in controls versus 18,664 ± 1,738 μm^2^ in DKD, *P* = 0.042), with a dramatic increase in glomerular APOB (Figure 2, A and B; Supplemental Figure 1; and Supplemental Table 1), indicating that non-HDL-associated lipid particles accumulate within the glomeruli under diabetic conditions. Furthermore, lipids appeared to accumulate intracellularly as perilipin 2 (a lipid droplet marker that is increased with lipid accumulation) staining was also increased in the setting of diabetes (Figure 2C).

### Silencing APOC3 reduces TRL accumulation in the kidney.

To test the mechanistic role of APOC3 and TRLs on DKD, we turned to a mouse model of type 2 diabetes and kidney disease. Leptin deficiency (OB) on the black and tan, brachyury (BTBR), background results in human-like DKD (20) and in the setting of silencing the LDL receptor (LDLR), human-like dyslipidemia with elevated levels of TRLs and atherosclerosis (21). The effect of APOC3 has been previously tested in a mouse model of DKD without human-like dyslipidemia. Wild-type (WT) mice, unlike humans, have very low levels of non-HDLs and, therefore, carry the majority of their APOC3 on HDL. Thus, silencing APOC3, under those circumstances, would primarily affect HDL-associated APOC3. Attie and colleagues inhibited APOC3 using an antisense oligonucleotide (ASO) in LDLR WT BTBR OB mice, which did not prevent DKD progression (22), consistent with the idea that TRL-associated APOC3 may play a role in DKD rather than HDL-associated APOC3.

Male and female BTBR WT and OB mice were treated with an LDLR ASO to induce dyslipidemia with elevated TRLs or a control ASO (cASO) for 14 weeks while being placed on an atherogenic diet (Figure 2D; Supplemental Figure 2, A and B; and Supplemental Table 2). Plasma APOC3 levels were elevated in OB mice, particularly in the setting of LDLR deficiency (Figure 2D), consistent with studies in humans demonstrating that people with insulin resistance have elevated levels of plasma APOC3, in part due to increased hepatic production (23).

To test the role of APOC3, mice within the cASO and the LDLR ASO groups received either an APOC3 ASO or a cASO (Figure 2D). As expected and consistent with previous work (9, 24), APOC3 ASO was excellent at suppressing the hepatic expression of *Apoc3* mRNA and plasma APOC3 levels (Figure 2E and Supplemental Figure 2C). Importantly, but consistent with the initial description of the APOC3 ASO (24), silencing APOC3 did not result in increased hepatic triglyceride accumulation (Supplemental Figure 2D).

Targeting the LDLR resulted in a dramatic increase in plasma triglycerides, total cholesterol, and VLDL and LDL in both WT and OB mice treated with the LDLR ASO for 14 weeks (Figure 2, F and G; Supplemental Figure 2, A and B; and Supplemental Table 2). As expected, APOC3 ASO dramatically reduced total and VLDL triglycerides and VLDL-cholesterol (Figure 2F and Supplemental Figure 2, A and B) but did not have a dramatic effect on total cholesterol (Figure 2G and Supplemental Table 2), suggesting that the main effect of the APOC3 ASO was on larger TRLs.

Leptin deficiency was associated with elevated blood glucose levels as early as 2 weeks after the initiation of the study (Supplemental Table 2). At that point, blood glucose was similar between the control and APOC3-treated OB mice, but starting at 4 weeks, leptin-deficient mice treated with the APOC3 ASO had significantly reduced blood glucose levels. This effect was more pronounced in the mice treated with the LDLR ASO, as they tended to have more exaggerated hyperglycemia than the control-treated OB mice (Figure 2H and Supplemental Table 2). The improvement in glycemic control was associated with a reduced fasting blood glucose and reduced glucose excursion in response to an intraperitoneal glucose tolerance test but not an improved response to insulin or to a pyruvate challenge or changes in expression of key genes in gluconeogenesis (Supplemental Figure 3). Rather, silencing APOC3 increased pancreatic islet insulin staining in OB mice treated with APOC3 ASO (Supplemental Figure 3I).

**Figure 3:**
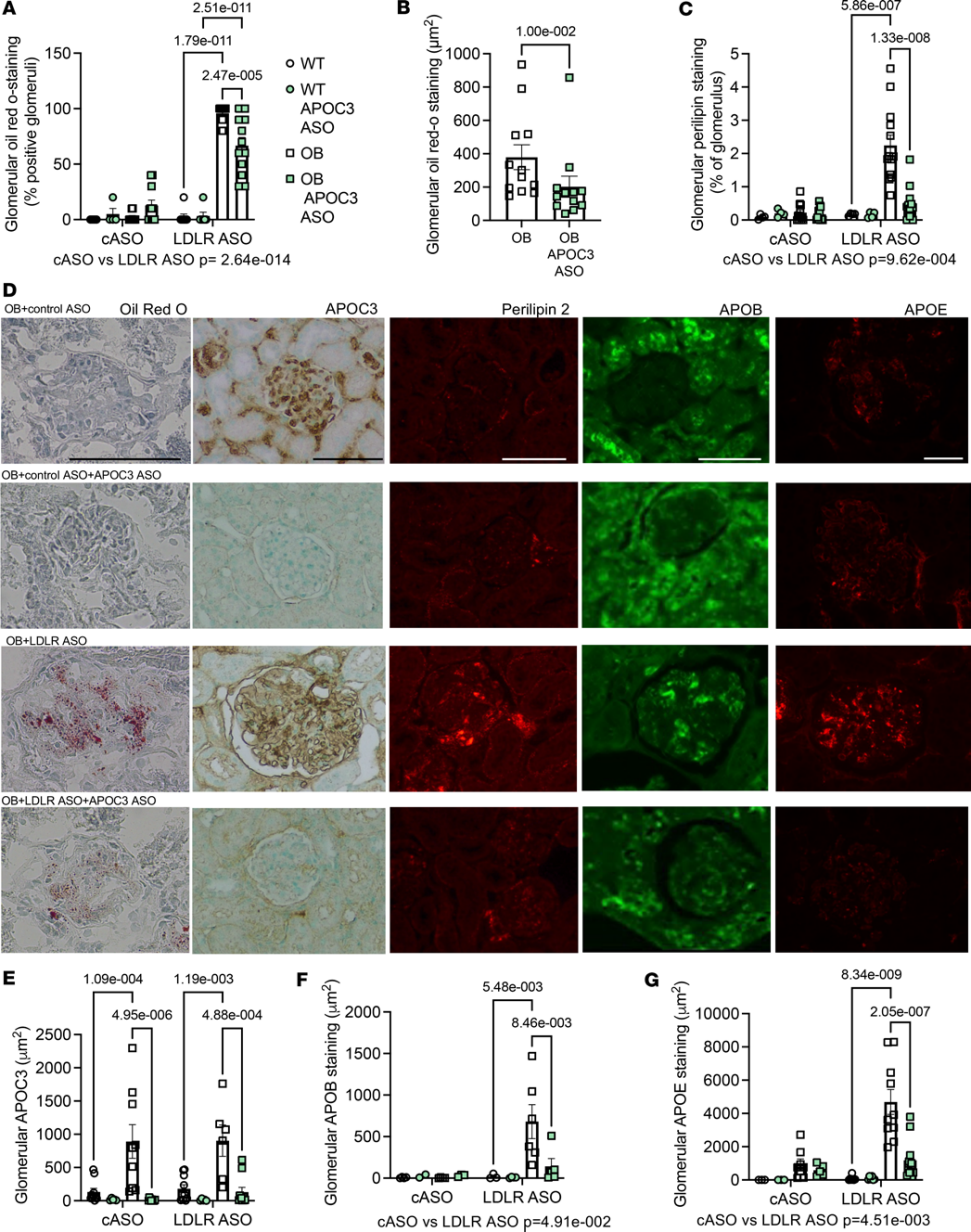
APOC3 ASO suppresses glomerular TRL accumulation. Mice were treated as in Figure 2. (**A**) Glomerular Oil Red O staining expressed as the overall percentage of positive glomeruli (independent of the extent of staining within a positive glomerulus; *N* = 5, 4, 9, 11, 8, 6, 12, 12). (**B**) Extent of glomerular Oil Red O staining in positive glomeruli (*N* = 12). (**C**) Extent of glomerular perilipin 2 staining expressed as percentage of glomerular area (*N* = 4, 4, 11, 12, 4, 4, 13, 11). (**D**) Representative images from cASO- or LDLR ASO–treated mice with or without the APOC3 ASO. WT displayed minimal staining for any of the lipid markers. See Supplemental Figure 4 for negative controls. (**E**) Glomerular APOC3 area (*N* = 10, 5, 9, 10, 10, 5, 6, 10). (**F**) Glomerular APOB area (*N* = 4, 2, 4, 4, 3, 3, 4, 6, 5). (**G**) Glomerular APOE area (*N* = 4, 4, 8, 5, 8, 6, 10, 12). Data expressed as mean ± SEM. Data were analyzed by 2-way ANOVA followed by Tukey’s multiple comparisons test. The text under the graph indicates the overall significance between cASO and LDLR ASO groups. The scale bar indicates 100 μm.

In the absence of LDLR deficiency, we observed very little neutral lipid accumulation in the kidney, as determined by Oil Red O staining or lipid droplet accumulation, as assessed by perilipin 2 staining, within the glomerulus in diabetes (Figure 3, A–D). However, in the setting of LDLR ASO treatment and diabetes, we observed a dramatic increase in Oil Red O staining and perilipin 2 staining (Figure 3, A–D), consistent with the findings from human diabetic nephropathy, providing evidence that the accumulating lipids are non-HDL-associated lipids. This increase in lipid accumulation was reduced in OB mice treated with APOC3 ASO. To identify what types of lipoproteins are accumulating and whether APOC3 silencing reduced glomerular lipoprotein accumulation, we stained the kidneys with antibodies against APOC3, APOB, and APOE (Figure 3, E–G, and Supplemental Figure 4A). Leptin deficiency resulted in a dramatic increase in glomerular APOC3 accumulation, and as expected, based on the circulating levels of APOC3, APOC3 ASO treatment completely blocked APOC3 accumulation in OB mice in the kidney (Figure 3E). Surprisingly, there was no difference between the mice treated with the control and the LDLR ASO, suggesting that both HDL-associated and non-HDL-associated APOC3-containing particles accumulate in the glomeruli under diabetic conditions. Alternatively, the APOC3 staining could reflect accumulation of lipid-free APOC3.

In contrast, in the setting of LDLR deficiency, there was a dramatic increase in both APOB and APOE accumulation in leptin-deficient mice, suggesting that non-HDL (APOB positive) particles indeed accumulated in the glomeruli (Figure 3, F and G). Importantly, APOC3 ASO treatment abolished APOB and APOE staining, arguing that silencing APOC3 reduces circulating TRLs and thus reduces the glomerular accumulation of TRLs. These data also imply that adding human-like dyslipidemia better mimics the lipid accumulation seen in human diabetic nephropathy.

### Reducing TRLs hampers DKD progression.

**Figure 4:**
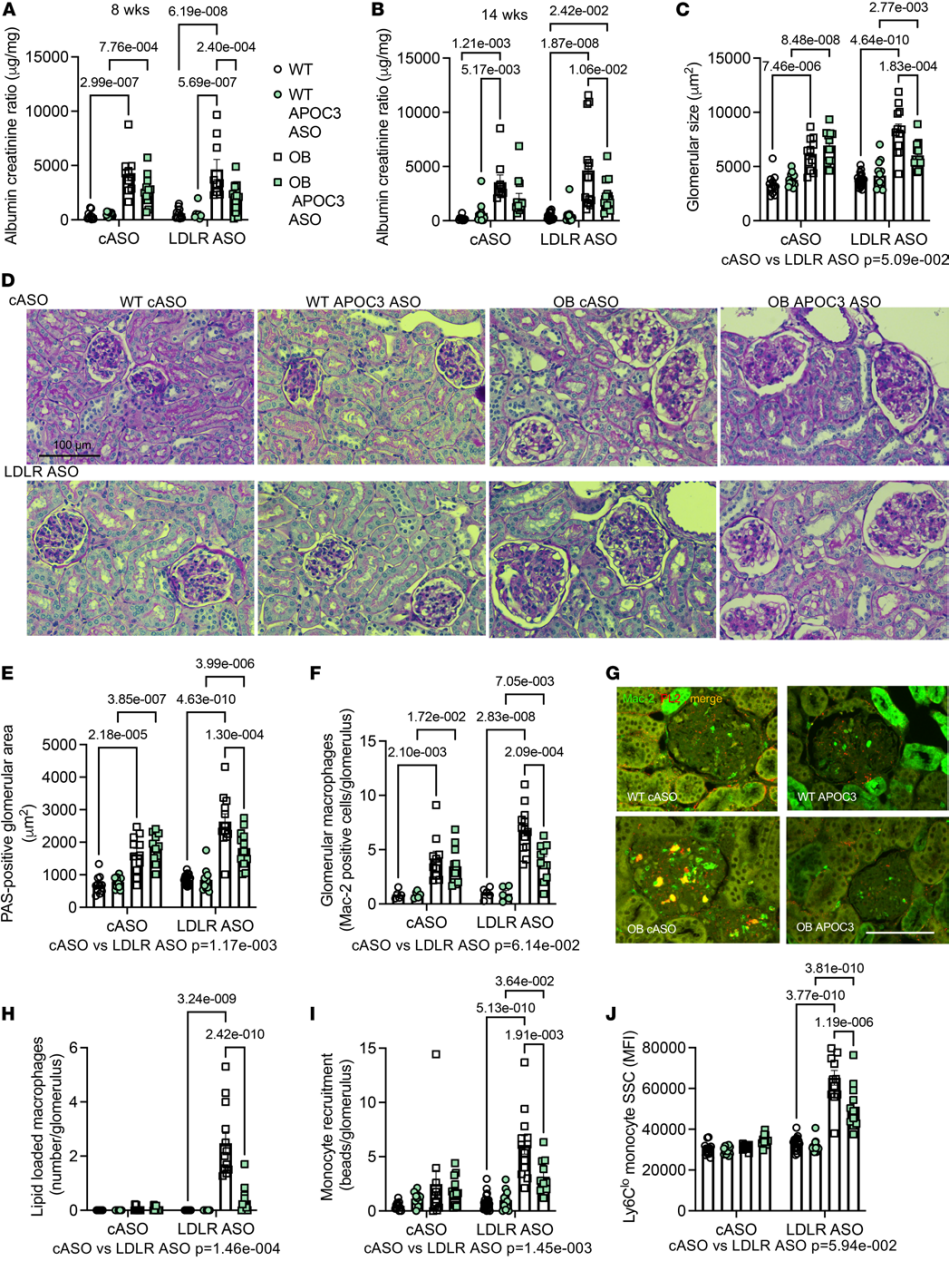
Silencing APOC3 hampers DKD progression. Mice were treated as in Figure 2, and urine was collected at 8 weeks (**A**) and 14 weeks (**B**). (**A**) Urine albumin-to-creatinine ratio at 8 weeks. (**B**) Urine albumin-to-creatinine ratio at 14 weeks. For *N*, urinary albumin, and creatinine values, please see Supplemental Table 3. (**C**) Glomerular size based on periodic acid–Schiff (PAS) staining. (**D**) Representative images of PAS staining. (**E**) Extent of PAS-positive glomerular area. (**F**) Glomerular Mac-2 staining (*N* = 5, 5, 10, 12, 5, 5, 13, 11). (**G**) Representative glomerular Mac-2 (green) and perilipin 2 (red) staining images from mice treated with the LDLR ASO. (**H**) Enumeration of perilipin 2^+^ glomerular macrophages (indicating lipid loading; *N* same as **F**). (**I**) Monocyte recruitment based on the number of yellow-green bead-labeled monocytes per glomerulus. For a representative image, please see Supplemental Figure 5J. (**J**) Side scatter (SSC) in Ly6C^lo^ monocytes indicates lipid loading. Data expressed as mean ± SEM. Data were analyzed by 2-way ANOVA followed by Tukey’s multiple comparisons test. The text under the graph indicates the overall significance between cASO and LDLR ASO groups. *N* as indicated in Figure 2D, unless otherwise noted. The scale bar indicates 100 μm.

We analyzed urinary albumin excretion throughout the study to evaluate whether APOC3 plays a role in DKD progression. Previous studies in which APOC3 was silenced in LDLR WT mice did not reveal a protective effect of APOC3 ASO treatment (22). Consistent with those data, we did not observe a significant effect of APOC3 ASO in nondiabetic or diabetic BTBR mice on albuminuria at 8 or 14 weeks without LDLR deficiency (Figure 4, A and B). Interestingly, in the setting of elevated TRLs induced by LDLR deficiency and diabetes, silencing APOC3 significantly reduced albuminuria at both 8 and 14 weeks (Figure 4, A and B, and Supplemental Table 3). This was also associated with an overall effect of APOC3 on plasma blood urea nitrogen (overall effect APOC3 ASO using 2-way ANOVA *P* = 0.03). The protective effect of APOC3 silencing was corroborated by the histological findings that APOC3 ASO prevented glomerular hypertrophy and glomerular matrix expansion as evident by PAS-positive matrix deposition (Figure 4, C–E) and silver methenamine (Supplemental Figure 3J). This protective effect of APOC3 ASO was not seen in the control ASO-treated mice, suggesting that blocking the glomerular accumulation of TRLs rather than reducing APOC3 on HDL is protective against DKD. Furthermore, APOC3 deposition correlated with glomerular size on a per-glomerulus basis, in LDLR-deficient OB mice, but not in OB mice without LDLR deficiency (Supplemental Figure 3K).

Podocytes, as assessed by Wilms’ tumor-1 staining, were reduced in OB mice. Although LDLR deficiency significantly worsened podocyte density, APOC3 ASO treatment did not statistically significantly alter their numbers (Supplemental Figure 4A).

We have previously demonstrated that adding a more human-like dyslipidemia with elevated LDL and TRLs augments the glomerular macrophage accumulation (21). Consistent with that finding, we observed that LDLR deficiency augmented macrophage accumulation (Figure 4F), which was reduced in leptin-deficient mice treated with the APOC3 ASO. Furthermore, a significant proportion of these macrophages were lipid loaded, as assessed by perilipin 2 staining (Figure 4, G and H), but only in the setting of diabetes and LDLR deficiency. In fact, in these mice, approximately 50% of the total glomerular perilipin 2 stain colocalized with the macrophage marker, and about 40% of macrophages were lipid loaded in OB mice with LDLR deficiency. This was abolished in mice treated with APOC3 ASO (Figure 4H). This increase in macrophage infiltration was associated with augmented plasma IL-18 levels, a cytokine often associated with macrophage inflammation, whereas liver-derived pro-inflammatory markers were unaltered (Supplemental Table 4). In addition to macrophage lipid loading, OB mice with LDLR deficiency displayed lipid droplet accumulation in tubular cells (Supplemental Figure 4).

To investigate if the macrophage accumulation was due to increased infiltration of monocytes, yellow-green (YG) latex beads were used to label circulating monocytes 4 days before euthanasia (25). Like humans, mice have 2 main monocyte populations, most often separated based on their expression of Ly6C (Supplemental Figure 5, A–H). Leptin deficiency was not associated with an increase in total white blood cells. Still, diabetes resulted in an increase in total monocytes and neutrophils unaffected by either LDLR deficiency or APOC3 ASO treatment. Both the Ly6C^hi^ and the Ly6C^lo^ populations were elevated in the setting of diabetes. Consistent with previous publications (25), injection of beads without depleting existing monocytes primarily labels Ly6C^lo^ monocytes. Approximately 20% of circulating Ly6C^lo^ monocytes were bead labeled, while less than 1% of Ly6C^hi^ monocytes were marked with YG beads (Supplemental Figure 5, G and H). In the setting of LDLR deficiency, diabetes resulted in a marked increase in the recruitment of these bead-labeled Ly6C^lo^ monocytes, which was diminished in mice treated with APOC3 ASO (Figure 4I). Notably, these Ly6C^lo^ monocytes appeared to be lipid loaded in circulation, as they displayed increased side scatter (Figure 4J), a surrogate marker for lipid loading (26). Reducing APOC3 prevented this increase in side scatter, which was not observed in Ly6C^hi^ monocytes (Supplemental Figure 6A). This suggests that elevated circulating levels of TRLs and concomitant TRL accumulation in the glomeruli increase the recruitment of monocyte-derived macrophages, some of which are already lipid loaded, resulting in augmented renal injury.

### Glomerular accumulation of lipids drives monocyte recruitment via increased endothelial ICAM1 expression.

**Figure 5:**
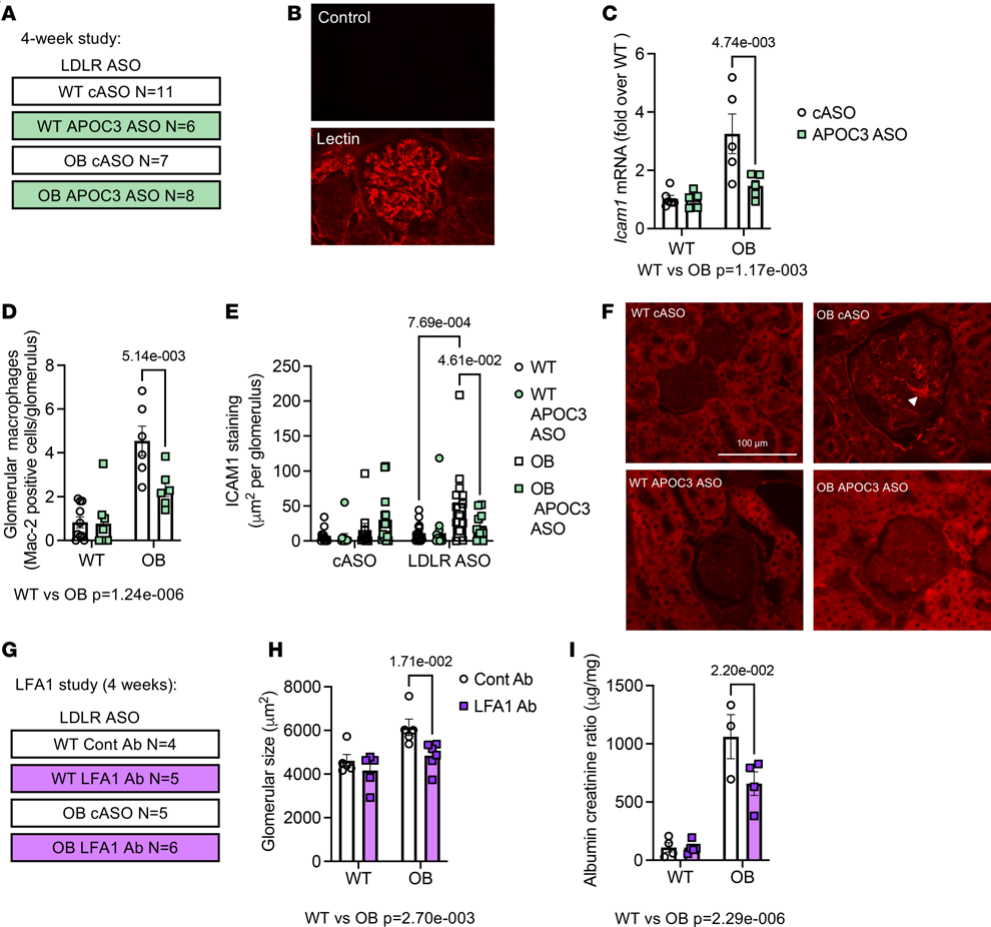
Blocking ICAM1/lymphocyte function-associated antigen 1 interaction reduces DKD. (**A**) Schematic of the study plan (4-week study). Briefly, WT and leptin-deficient OB mice were treated with LDLR ASO and either a cASO or an ASO to APOC3. Mice were then placed on a high-fat diet for 4 weeks. (**B**) Representative staining of lectin labeling of endothelial cells in the kidney. (**C**) *Icam1* mRNA in isolated kidney cortex endothelial cells. (**D**) Glomerular Mac-2 staining. (**E**) Glomerular ICAM1 staining from the 14-week study (described in Figure 2). (**F**) Representative ICAM1 staining. The white arrow indicates endothelial ICAM1 staining in the glomerulus. (**G**) Schematic of the study plan to test the role of ICAM1/LFA1 interaction (LFA1 study). Briefly, WT and leptin-deficient OB mice were treated with LDLR ASO and either a control antibody or ICAM1 blocking antibody while on a high-fat diet for 4 weeks. (**H**) Glomerular size based on PAS staining. (**I**) Urine albumin-to-creatinine ratio from urine collected at week 4 (*N* = 4, 5, 3, 4). Data expressed as mean ± SEM. Data were analyzed by 2-way ANOVA followed by Tukey’s multiple comparisons test. The text under the graph indicates the overall significance between cASO and LDLR ASO groups. *N* as indicated in **A** and **G**, unless otherwise noted. The scale bar indicates 100 μm.

Monocyte recruitment into tissues is a highly regulated process that depends on monocytes and endothelial cells’ expression of adhesion molecules and respective ligands (27). To better understand how APOC3 silencing might impact monocyte recruitment, monocyte surface expression of molecules known to regulate adhesion and migration was evaluated after 4 weeks of diabetes, all in the setting of LDLR ASO (Figure 5A and Supplemental Table 5). Lymphocyte function-associated antigen 1 (LFA1) was increased on Ly6C^lo^ monocytes but not on Ly6C^hi^ monocytes, while CX3CR1 and CD49D were unchanged on both monocyte populations (Supplemental Figure 6B and Supplemental Table 5). APOC3 ASO did not alter the expression of any adhesion molecules, so we turned our attention to the endothelial cells as potential regulators of monocyte recruitment. To isolate renal endothelial cells, we used an in vivo labeling approach that relies on lectin from *Lycopersicon esculentum* binding to the endothelial cells (28). Indeed, we observed that lectin labeling mainly labeled endothelial cells, including those in the glomerulus (Figure 5B). We then used this approach to isolate renal cortex endothelial cells. ICAM1 is one of the key ligands for LFA1 and has previously been implicated in DKD (29–31). Consistent with that, we observed that endothelial cell expression of *Icam1* mRNA was increased in OB mice, which was reverted to WT levels in mice treated with APOC3 ASO (Figure 5C). The increased endothelial cell *Icam1* expression in OB mice and the corresponding reduction with APOC3 ASO correlated with increased and diminished glomerular macrophage accumulation, respectively (Figure 5D), consistent with our previous findings (Figure 4).

Endothelial cell *Vcam1*, *Ccl2*, and *Cx3cl1* expression trended in the same direction but did not reach statistical significance (Supplemental Figure 6, C–E). In addition, *Plin2* expression was elevated in endothelial cells that were isolated from OB mice (Supplemental Figure 5F) and trended down in endothelial cells from mice treated with APOC3 ASO, suggesting that lipid droplets form in endothelial cells and can be lowered by APOC3 ASO treatment.

To assess whether ICAM1 protein was increased and if it was indeed altered in the glomeruli with diabetes and APOC3 ASO, kidney sections from the original study were stained for ICAM1. Consistent with the endothelial mRNA experiment data, OB mice displayed significantly more glomerular ICAM1 staining than WT mice under LDLR ASO conditions, which was abrogated with APOC3 ASO treatment (Figure 5, E and F).

To establish a role of LFA1/ICAM1 in the DKD progression, WT and OB mice were treated with either a control antibody or an LFA1 blocking antibody for 4 weeks while being treated with the LDLR ASO. At the end of the 4 weeks, blocking LFA1 did not affect blood glucose, plasma triglycerides, or cholesterol (Supplemental Table 6), but blocking LFA1 reduced glomerular size and albuminuria in OB mice (Figure 5, G–I).

Together, this indicates that elevated TRLs are associated with increased endothelial cell ICAM1 expression, allowing for increased monocyte recruitment, which mediates some of the deleterious effects of diabetes on the glomerulus.

### APOC3 inhibition in the absence of improvement in glycemia still reduces DKD.

**Figure 6:**
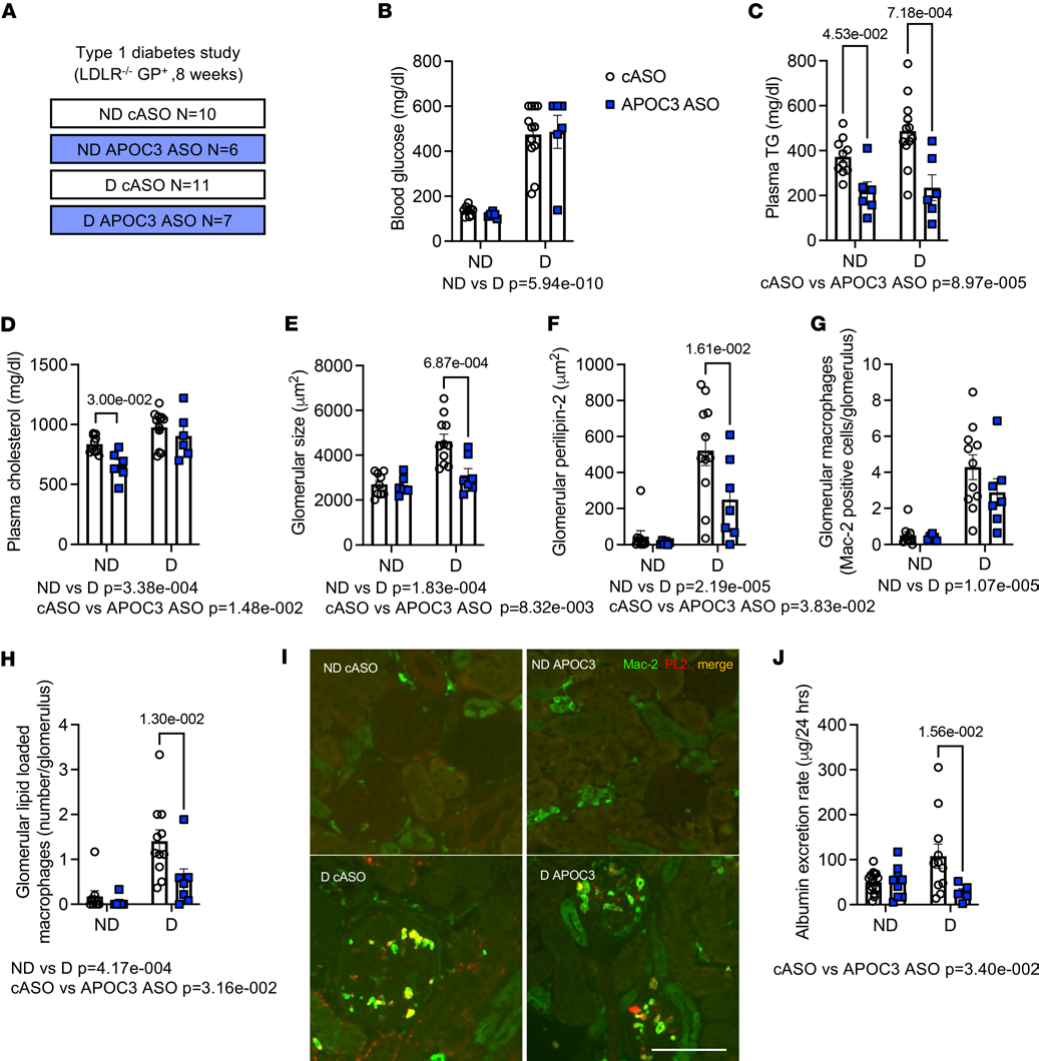
APOC3 inhibition in the absence of improvement in glycemia still reduces DKD. (**A**) Schematic of the study plan (type 1 diabetes study). Briefly, male *LDLR^–/–^ Gp^+^* were injected with saline (nondiabetic: ND) or lymphocytic choriomeningitis virus (LCMV) to induce diabetes (**D**) and treated with either a cASO or an ASO to APOC3 for 10 weeks while on the high-fat diet. (**B**) Blood glucose at 10 weeks. (**C**) Plasma triglycerides (TG) at 10 weeks. (**D**) Plasma cholesterol at 10 weeks. (**E**) Glomerular size. (**F**) Glomerular perilipin 2 staining. (**G**) Glomerular Mac-2 staining. (**H**) Enumeration of perilipin 2^+^ glomerular macrophages (indicating lipid loading). (**I**) Representative images of Mac-2 (green) and perilipin 2 staining (red). (**J**) Urinary albumin excretion. Data expressed as mean ± SEM. Data were analyzed by 2-way ANOVA followed by Tukey’s multiple comparisons test. The text under the graph indicates the overall significance between cASO and LDLR ASO groups. *N* as indicated in **A**. The scale bar indicates 100 μm.

Since APOC3 silencing reduced blood glucose, and improved glycemia is associated with improved kidney disease, we tested the effect of APOC3 silencing in a model in which APOC3 ASO does not reduce blood glucose (9). Type 1 diabetes was induced in LDLR-deficient mice using viral mimicry and maintained on the same high-fat diet as the BTBR mice (Figure 6A). Consistent with our previous data (9), APOC3 ASO did not affect blood glucose (Figure 6B) but had a dramatic effect on plasma triglycerides and a smaller effect on total cholesterol (Figure 6, C and D). Importantly, APOC3 ASO treatment reduced diabetes-associated glomerular hypertrophy, glomerular perilipin 2 staining, macrophage accumulation, and lipid-loaded macrophage accumulation (Figure 6, E–I). Although diabetes does not result in dramatic albuminuria in this model, silencing APOC3 prevented the increase in urinary albumin excretion associated with diabetes (Figure 6J), suggesting that the improvement observed with APOC3 ASO is not due to an improvement in glycemia. To further strengthen the postulate that APOC3 plays a role in DKD and that it is liver-derived APOC3, we treated BTBR WT and OB mice with a newer generation of the APOC3 ASO, which has a higher hepatic specificity because of an *N*-acetylgalactosamine (GalNAc) modification, allowing for a lower dose, together with the LDLR ASO. The mice were also on a semipurified low-fat diet (9, 32) instead of the aggressive atherosclerotic diet, for 12 weeks. Consistent with the previous findings, silencing APOC3 reduced plasma triglycerides and blood glucose, which was associated with a reduction in glomerular lipid accumulation, glomerular hypertrophy, and albumin/creatinine ratio in OB mice (Supplemental Figure 7 and Supplemental Table 7).

### Silencing APOC3 reduces atherosclerosis.

**Figure 7:**
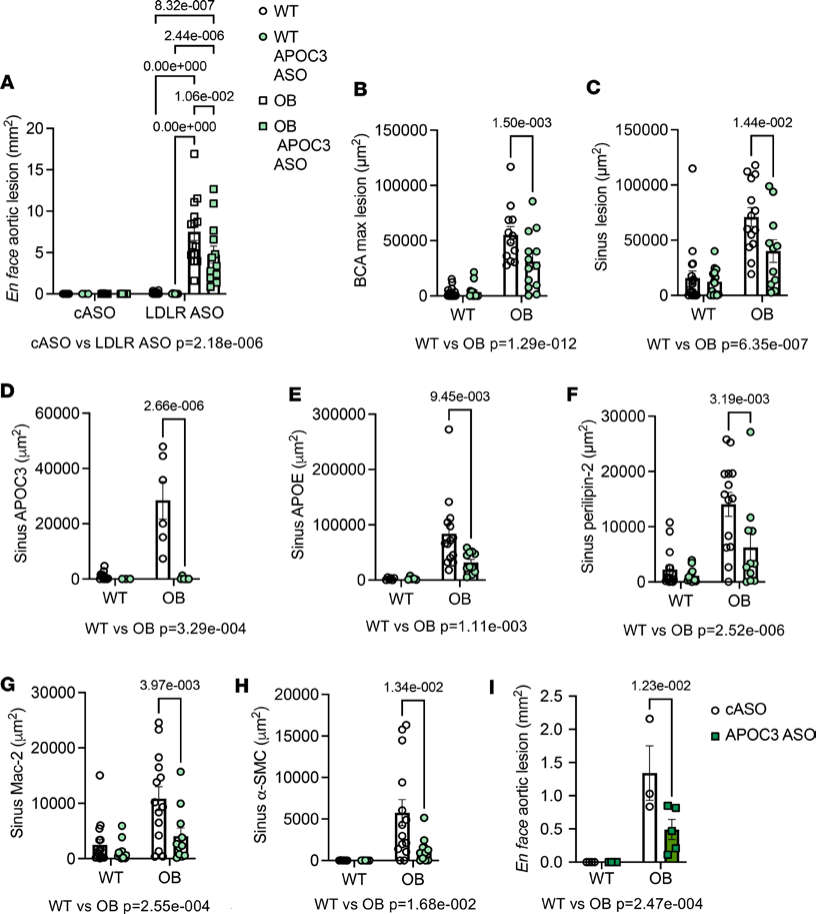
Silencing APOC3 reduces atherosclerosis. (**A**–**F**) (*N* = 7, 3, 5, 5 for cASO group; *N* as indicated in Figure 2D for LDLR ASO). Atherosclerosis was analyzed at the aorta, aortic sinus, and brachiocephalic artery (BCA) from the 14-week study on a high-fat diet (described in Figure 2). Examples of lesions can be found in Supplemental Figure 8. (**A**) En face aortic lesions as assessed by Sudan IV staining. (**B**) Maximal lesion area in the BCA. (**C**) Sinus lesion at the site where all 3 leaflets are first apparent. (**D**) Sinus APOC3 staining. (**E**) Sinus APOE staining. (**F**) Sinus perilipin 2 staining. (**G**) Sinus Mac-2 staining. (**H**) Sinus α–smooth muscle actin (SMC). (**I**) En face aortic lesions as assessed by Sudan IV staining from the 12-week study with the GalNAc-modified APOC3 and the low-fat diet (*N* = 4, 4, 3, 5). Data expressed as mean ± SEM. Data were analyzed by 2-way ANOVA followed by Tukey’s multiple comparisons test. The text under the graph indicates the overall significance between cASO and LDLR ASO groups.

We have previously demonstrated that APOC3 plays an important role in type 1 diabetes–accelerated atherosclerosis (9). Since underlying kidney disease is a strong risk factor for cardiovascular disease and kidney disease augments plasma levels of APOC3, we asked whether silencing APOC3 would have the same protective effect in a model of type 2 diabetes that also has underlying kidney disease. To this end, atherosclerosis was analyzed at 3 sites (aorta, brachiocephalic artery, and aortic sinus). As expected, the absence of LDLR deficiency did not result in atherosclerosis despite the atherosclerotic diet. However, in the presence of LDLR deficiency, both WT and OB mice developed atherosclerosis in the aorta, though OB mice developed significantly more, which was suppressed by APOC3 ASO treatment (Figure 7A and Supplemental Figure 8). This was consistent in the brachiocephalic artery and aortic sinus (Figure 7, B and C). The improvement in lesional phenotype was associated with reduced staining for APOC3, APOE, and perilipin 2, suggesting reduced accumulation of pro-atherogenic lipoproteins (Figure 7, D–F). There were also reductions in macrophage accumulation and smooth muscle cell accumulation with APOC3 ASO treatment (Figure 7, G and H), indicating an overall less advanced lesional phenotype. The reduction in atherosclerosis in diabetes with decreasing APOC3 levels was also observed in the setting of GalNAc-modified APOC3 ASO treatment (Figure 7I).

## Discussion

Here, we demonstrate that plasma APOC3 can predict renal functional loss in people with type 2 diabetes. Together with the recently published report from the FinnDiane study (8), our finding highlights the possibility that TRLs may contribute to the progression of kidney disease in diabetes. This is further supported by a large observational study, where triglycerides, but not total cholesterol or LDL-cholesterol, were associated with advanced kidney disease (33).

Mechanistically, we demonstrate that TRLs accumulate in the kidney in the setting of DKD and that inhibiting this hampers DKD progression. Based on our experiments with the hepatically targeted APOC3, we postulate that the majority of the TRLs that are accumulating in the kidney are liver derived. However, we cannot disregard that some of the apolipoproteins can be produced locally in proximal tubular cells and macrophages, which are known to be able to produce APOB, APOC3, and APOE, respectively (13, 34).

The notion that it is the TRLs that accelerate renal injury is consistent with our findings in the absence of LDLR deficiency, where silencing APOC3 had no effect. These data are very much in line with the finding by Attie et al., who described no effect of APOC3 inhibition in WT and OB mice without LDLR deficiency (22). Furthermore, this is supported by the notion that transgenic overexpression of APOC3, which results in elevated levels of TRLs (35), augments early DKD in a model of type 1 diabetes, partly via elevated renal inflammation (36). APOC3 deficiency, however, did not protect against diabetes-induced injury (36), again in the absence of elevated TRLs.

How do reduced levels of APOC3 slow the progression of DKD? Based on our findings, we postulate that elevated TRLs induce endothelial cell activation, which in turn drives monocyte recruitment to the glomerulus. Ample data from preclinical models in the literature suggest that excess accumulation of macrophages in the kidneys contributes to renal injury, and consistent with our proposed mechanism, ICAM1-deficient diabetic mice have reduced accumulation of glomerular macrophages, reduced glomerular hypertrophy, and improved diabetes-induced albuminuria (30). Additionally, there is some genetic evidence that ICAM1 could contribute to DKD in humans (37), where a polymorphism in the *ICAM1* gene, which might render it more active in its binding to LFA1, may contribute to DKD progression (38). Together, this most likely results in functional changes to the glomerular filtration unit, resulting in augmented albuminuria. Several studies have indicated changes in the matrix (glycocalyx) surrounding the endothelial cells can affect the filtration and, thus, albuminuria. However, we cannot discount the fact that other cells in the kidney are likely also affected by the changes in circulating lipids. For example, we observe that tubular cells contain lipid droplets, which is reversed by APOC3 ASO treatment, and defective lipid metabolism in tubular epithelial cells has been shown to contribute to kidney disease (39). Furthermore, podocytes are known to be sensitive to changes in cholesterol, and altering their ability to handle cholesterol contributes to DKD (13, 40).

In addition to the reductions in TRLs, silencing APOC3 reduced blood glucose in a type 2 diabetes model, which has also been reported in humans (41). Numerous studies have indicated the beneficial effect of blood glucose lowering in people with diabetes on kidney disease progression (42–44), suggesting perhaps that the improvement in kidney disease could, in part, be due to improved glycemia. To address this, we turned to a model of type 1 diabetes in which the pancreatic β cells are lost; thus, APOC3 silencing does not improve blood glucose. Although the kidney injury is less pronounced in this model, we still observed an improvement in markers of kidney disease with decreasing TRLs, arguing that TRLs are indeed involved.

Whether the effect of silencing APOC3 is related to the direct effects of APOC3 enrichment on TRL particles or the lipids from said particles is unclear at this point. Lipid-free, but not lipid-bound, APOC3 has been shown to induce monocyte inflammasome activation (45, 46). Although very little lipid-free APOC3 can be found in circulation (46), perhaps, locally, especially in the kidney, which is known to contribute to the clearance of APOC3, lipid-free APOC3 could be found. Others have demonstrated that TRLs can directly induce inflammatory changes in endothelial cells, including upregulating adhesion molecules (47, 48). Some studies suggest that APOC3 mediates these effects, whereas others do not (47, 48). Additionally, altering TRL may affect other cells, which in turn affect the kidney.

Finally, we demonstrate, similar to a model of type 1 diabetes (9), that silencing APOC3 reduces atherosclerosis associated with diabetes, even in the presence of kidney disease. Together, this suggests that lowering plasma APOC3 might be a way to target both atherosclerotic cardiovascular disease and DKD.

## Methods

Additional methods can be found in Supplemental Methods. Key reagents are listed in Supplemental Table 8.

### Sex as a biological variable

The VADT includes mainly men. Thus, sex was not addressed as a biological variable. Both male and female mice were used in this study (see Supplemental Tables 1, 2, and 5–7). No differences were noted between the sexes, and the data were therefore combined.

### Plasma APOC3 and renal disease progression in VADT

Plasma APOC3 concentrations were measured in 1,722 available baseline plasma samples from the VADT (ClinicalTrials.gov NCT00000620). A detailed description of the VADT has been previously published (19). VADT enrolled a total of 1,791 US veterans with poorly controlled type 2 diabetes. The primary treatment goal of the VADT was to achieve a 1.5% difference in hemoglobin A1c between those randomized to intensive versus standard therapy while achieving optimal levels of other conventional cardiovascular risk factors. In the VADT, intensive glycemic control was not associated with a reduction in macrovascular or microvascular events compared with standard glycemic therapy (18). Serum and urine creatinine and urine albumin were measured annually by the VADT central laboratory at Tufts University. Plasma hemoglobin A1c, glucose, cholesterol, triglycerides, and HDL-cholesterol concentrations were measured using standard enzymatic methods on a Hitachi 911 analyzer, with reagents obtained from Roche Diagnostics. eGFR was calculated by the Chronic Kidney Disease Epidemiology Collaboration equation (49). Urine albumin excretion was estimated by ACR. Participants with eGFR ≥ 120 mL/min/1.73 m^2^, indicating glomerular hyperfiltration, and those with plasma triglycerides > 500 mg/dL, indicating hypertriglyceridemia, were excluded from the analyses. Total plasma APOC3 concentrations were measured by immunoturbidimetric method (Kamiya Inc.) on the Abbott Architect c8000 automatic analyzer. The primary outcome was the first occurrence of significant RFL, defined as a 40% or greater decline in eGFR from baseline, sustained through at least 2 subsequent visits. Large meta-analyses showed that 40% RFL is an excellent surrogate for progression into end-stage renal disease and fatal kidney disease and has been recommended as an endpoint in cohorts with relatively preserved kidney function and a limited number of serious events over the follow-up period (50, 51).

### Analysis of APOB and perilipin 2 staining in diabetic nephropathy

Renal biopsies were obtained from pathology-verified healthy nondiabetic controls and people with diabetic nephropathy. The stage of diabetic glomerular disease in the biopsy was assessed by a practicing renal pathologist using a published scheme (52). APOB and perilipin 2 staining were carried out as described below.

### Mouse model of kidney disease and atherosclerosis

Male and female BTBR WT and BTBR mice homozygous for the leptin deficiency mutation (*Lep^ob^*; OB) were used in this study. The study was initiated when the mice were 4–5 weeks of age.

#### Fourteen-week study.

At that point, they were placed on a semipurified high-fat diet containing 40% calories from fat (high in saturated fatty acids from milk fat and cocoa fat), 40% calories from carbohydrates, and 1.25% added cholesterol and maintained for 5 or 14 weeks. Based on baseline glucose and weights, the mice were allocated into different experimental groups. The LDLR ASO (GalNAc modified to increased hepatic targeting) and a scrambled ASO control were injected intraperitoneally once a week for the duration of the study at 5 mg/kg/w to induce human-like dyslipidemia (53). To test the role of APOC3, a subset of mice was injected with APOC3 ASO at 50 mg/kg/w (not GalNAc modified) for the duration of the study. All ASOs were from Ionis Pharmaceuticals. Mice were monitored weekly for body weight, and blood glucose, cholesterol, and triglycerides were measured at 0, 2, 4, 8, and 14 weeks. Urine was measured in modified cages for urine collection at weeks 8 and 14.

#### Four-week study.

Mice were treated as above, except all mice were treated with the LDLR ASO and maintained for 4 weeks. Fifteen minutes before euthanasia, mice were sedated and injected with biotinylated *Lycopersicon esculentum–*derived lectin (Vector Laboratories, B-1175) to label endothelial cells. Kidney cortices were digested with collagenase 1 (Worthington, LS004196, 2 mg/mL) in the presence of the Polymerase 2 inhibitor Flavopiridol (final concentration 1 μM) twice for 15 minutes. Biotin-positive cells were then isolated using the EasySep Biotin Positive Selection Kit II (STEMCELL Technologies), and the isolated cells were washed and lysed for RNA extraction. One piece of one kidney was used to determine the specificity of the in vivo labeling. These were processed, sectioned, and stained for the presence of biotin. Body weight, blood glucose, plasma cholesterol, and triglycerides can be found in Supplemental Table 5.

#### LFA1 blocking study.

To address whether blocking LFA1 affects DKD, we carried out a 4-week study where WT and OB BTBR mice were maintained on a high-fat diet and LDLR ASO while treated with a blocking antibody against LFA1 (clone M17/4) or an isotype control antibody targeting trinitrophenol (a protein not expressed in mice) at 0.9 mg/mouse/w. The blood glucose, plasma cholesterol, and triglycerides were measured at 0 and 4 weeks (Supplemental Table 6). Urine was measured in modified cages for urine collection at 4 weeks. At the end of the study, kidneys were isolated and processed as described below.

#### Twelve-week study on a low-fat diet.

To rule out that the results were specific to the high-fat diet, we carried out a study where WT and OB BTBR mice were fed a semipurified low-fat diet (10% of calories from fat) (32) for 12 weeks while treated with a GalNAc-modified APOC3 ASO or cASO (10 mg/kg/w) and the LDLR ASO. Body weights, blood glucose, triglycerides, and cholesterol can be found in Supplemental Table 7.

#### Mouse model of type 1 diabetes and kidney disease.

The T cell–induced transgenic *LDLR^–/–^ Gp^+^* mouse model of type 1 diabetes–accelerated atherosclerosis has been described previously (32). These mice express the LCMV GP under the control of the insulin promoter. After the virus is injected, CD8^+^ T cells destroy β cells of the pancreas, inducing diabetes. At the onset of diabetes, the mice (all male) were switched to the same semipurified diet as the BTBR mice and maintained for 8 weeks. At the end of the study, kidneys were isolated and processed as described below.

### Analysis of plasma and fast protein liquid chromatography fractions

Blood glucose was measured in the saphenous vein blood by stick tests (OneTouch Ultra). As the glucometer does not go beyond 600 mg/dL, higher values were set to 600 mg/dL. Plasma cholesterol levels were determined by the Cholesterol Liquicolor (EKF Diagnostics), and plasma TGs were determined by Triglyceride Liquicolor. Plasma lipoprotein profiles were analyzed by size-exclusion fast protein liquid chromatography, as described previously (32). Plasma APOC3 and fast protein liquid chromatography fractional APOC3 levels were measured using an APOC3 ELISA from Abcam. Plasma IL-18, IL-6, TNF-α, TGF-β1, and IL-10 were measured using ELISAs from Invitrogen, Thermo Fisher Scientific.

### Flow cytometry of blood leukocytes

Mice were bled from the retro-orbital plexus under isoflurane sedation after 12 weeks of diabetes. EDTA was the anticoagulant. Total leukocytes were measured using an automated cell counter for mouse blood samples (Hemavet; Drew Scientific). For flow cytometric analysis, erythrocytes were lysed with an ammonium-chloride-potassium buffer and discarded. Leukocytes were stained using a fixable viability dye, CD45, CD115, and GR1 (clone 30-F11, AFS98, and RB6-8C5, respectively, eBioscience, now Thermo Fisher Scientific). Monocytes were identified as CD115-positive cells, and neutrophils were identified as CD115-negative GR1^hi^ cells. All analyses were performed with live, single, CD45-positive cells. The monocytes were further divided into GR1^hi^ (Ly6C^hi^) and GR1^lo^ (Ly6C^lo^) subpopulations. The cells were analyzed on a BD FACS RUO flow cytometer. Flow cytometric analysis was normalized to total white blood cell (WBC) counts and expressed as cells/mL of blood, assuming all WBCs are CD45-positive.

### Real-time PCR

Gene expression in the liver and isolated renal cortex endothelial cells was quantified by real-time PCR. RNA isolation and the real-time PCR protocol were performed as described previously (54). RNA was isolated using QIAGEN RNeasy or MACHEREY-NAGEL Nucleospin RNA Plus kits according to the manufacturer’s protocols. Real-time PCR was performed using the SYBR Green 1 detection method (Thermo Fisher Scientific). Cycle threshold values were normalized to *Rn18s* and presented as fold-change over control. Primer sequences are listed in Supplemental Table 9.

### Measurement of DKD

Similar to what we have done previously (21), urine was collected during a 4-hour fast. Urinary albumin was measured using a mouse albumin ELISA and normalized to urinary creatinine levels (Albuwell and Creatinine Companion, Exocell). Urinary volumes and total 24-hour urine and albumin secretion were calculated and presented in Supplemental Table 4. At the end of the study, the kidneys were cut longitudinally, embedded, and sectioned, similar to what we have done previously (21). One piece was fixed and embedded in paraffin; another was fixed and embedded in optimal cutting temperature for Oil Red O staining. Mac-2 immunostaining was performed after sodium citrate/Tween antigen retrieval (rat anti-mouse Mac-2 antibody: CL8942AP; Cedarlane) (21, 55). Mac-2^+^ glomeruli were counted in a minimum of 10 glomerular cross sections and expressed as the average number of cells per glomerulus. Lipid-loaded macrophages were identified as those Mac-2^+^ cells that were also perilipin 2–positive. Perilipin 2 staining was also quantified in the tubules surrounding the glomeruli. Podocytes were identified using p57 immunoreactivity (sc8298; Santa Cruz Biotechnology). Glomerular podocytes were counted, and their nuclear diameter was measured to estimate the total number of podocytes per glomerulus, as described by Venkatareddy et al. (56). PAS and silver methenamine stains were used to quantify mesangial expansion. Negative isotype controls (or serum controls) of the same concentrations or dilutions were used as controls. Examples of uncropped positive and negative control stains can be found in Supplemental Figure 4.

Circulating monocytes were labeled 4 days before euthanasia by injecting mice with Fluoresbrite green fluorescent (YG) plain microspheres (Polysciences Inc.) diluted 1:4 in sterile PBS. These microspheres were primarily taken up by Ly6C^lo^ monocytes, and YG beads were counted per glomerulus. For all analyses, 15–20 glomeruli were analyzed by an investigator masked to the experimental groups. When possible, image analysis was standardized using thresholding within areas of interest using ImageJ (NIH).

### Quantifying atherosclerosis and apolipoprotein staining

The aortic sinus was analyzed at 2 separate sites (90 μm apart), beginning when all 3 aortic valve leaflets appeared (57). Lesion macrophages were visualized by Mac-2 immunohistochemistry using a monoclonal rat anti-mouse Mac-2 antibody (CL8942AP at 1 μg/mL; Cedarlane). APOC3 immunohistochemistry was carried out with a rabbit polyclonal anti-APOC3 antibody generated by Ionis Pharmaceuticals (1:1,000 dilution). Immunohistochemistry for APOB and APOE was performed using a biotinylated goat anti-APOB antibody (R&D Systems, Bio-Techne, BAF3556; at 1:50) and a rabbit monoclonal anti-APOE antibody (Abcam, ab183597; at 1:2,000), respectively. α-SMA immunohistochemistry was performed using a rabbit anti–α-SMA antibody (Abcam, ab5694; at 1:1,000). For the APOB staining, the signal was enhanced by using Tyramide-Alexa Fluor 488 (Invitrogen) after incubation with streptavidin-HRP. All analyses were carried out by an investigator with experimental groups blinded.

### Statistics

Statistical analyses of the mouse data were performed using GraphPad Prism 9.4. We used 2-tailed unpaired Student’s *t* tests to compare differences when only 2 groups were compared. To compare more groups, we used a 2-way ANOVA. If there were 2 subgroups, a Bonferroni post hoc test was used, whereas Tukey’s post hoc test was used for subgroups of 4, as indicated in each figure legend. The overall statistical significance is listed below each graph (results of the 2-way ANOVA), whereas the lines represent the post hoc test results. D’Agostino-Pearson omnibus normality tests were performed to evaluate if the data were normally distributed. Statistical outliers were identified by the ROUT (*Q* = 1%) method. Statistical outliers are indicated in the figure legends. Data not normally distributed were analyzed by Kruskal-Wallis tests followed by Dunn’s multiple-comparison tests (multiple groups) or Mann-Whitney tests (2 groups).

Statistical analyses of the plasma APOC3 in the VADT study were conducted using SAS v9.4 (SAS Institute). All non-normally distributed continuous variables were natural log-transformed. Cox proportional hazard regression models assessed the association between baseline APOC3 measures and RFL. Proportional hazard assumptions were assessed by inspecting Kaplan-Meier curves for extreme quartiles of exposure measures and formally tested by cumulative sums of Martingale residuals with *P* values of the Kolmogorov-type supremum test. All models were first run-adjusted for glucose-lowering group assignment (model 1) and then adjusted for age, sex, race and ethnicity, diabetes duration, baseline hemoglobin A1c, history of hypertension, systolic blood pressure, eGFR, and proteinuria category (model 2), and then model 2 variables and plasma triglycerides (model 3). A *P* value of less than 0.05 was considered statistically significant.

### Study approval

All mouse experiments were performed in accordance with an approved University of Washington Institutional Animal Care and Use Committee protocol (protocol 3154-01). In adherence to the Declaration of Helsinki, all patients provided written informed consent for the collection of personal data prior to inclusion in the study with approval from the University of Washington (ethics review board no. 9950). The institutional review board approved all protocols and consent forms at each of the 20 participating sites for the VADT (18). All samples were deidentified and analyzed in a blinded manner.

### Data availability

Values for all data points in graphs are reported in the Supporting Data Values file.

## Author contributions

JEK designed and directed the study. FK and JC performed experiments and analyzed data. SA provided the human kidney sections. AEM provided advice and reagents. CEA provided reagents and advice. PR designed and directed the VADT study. JK directed the APOC3 assay and analyzed data in the VADT. All authors reviewed the manuscript and provided final approval for submission.

## Supplementary Material

Supplemental data

Supporting data values

## Figures and Tables

**Table 1:**
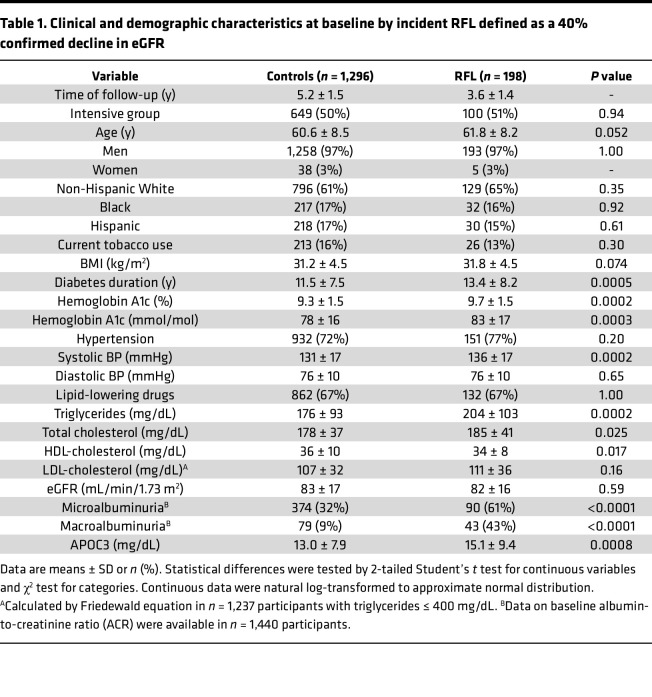
Clinical and demographic characteristics at baseline by incident RFL defined as a 40% confirmed decline in eGFR

## Version 1 05/14/2024

In-Press Preview

## Version 2 06/24/2024

Electronic publication
