# Supplementary material for: Elevated apolipoprotein C3 augments diabetic kidney disease and associated atherosclerosis in type 2 diabetes
Source: JCI Insight. 2024 May 14;9(12):e177268. doi: 10.1172/jci.insight.177268 (PMC11383354; doi:10.1172/jci.insight.177268)
Supplement: Supplemental data [file jciinsight-9-177268-s035.pdf]

Supplemental material for:
Elevated Apolipoprotein C3 augments diabetic kidney disease and associated atherosclerosis in type 2 diabetes

Jocelyn Cervantes[1#], Juraj Koska[2#], Farah Kramer[1], Shreeram Akilesh[3], Charles E Alpers[3], Adam E Mullick[4], Peter Reaven[2], Jenny E Kanter[1]

[1]Division of Metabolism, Endocrinology, and Nutrition, UW Medicine Diabetes Institute, University of Washington, Seattle, WA
[2] Phoenix VA Health Care System, Phoenix, AZ
[3]Department of Laboratory Medicine and Pathology, University of Washington, Seattle, WA
[4]Ionis Pharmaceuticals, Carlsbad, CA
#Contributed equally

Corresponding author:
Jenny E Kanter
UWMDI, University of Washington
South Lake Union Campus room F601,
750 Republican Street
Seattle, WA 98109
jenka@uw.edu
206-616-6095

**Supplemental Methods:**

*Glucose tolerance test*

Mice were fasted for 4-6 hours before injecting either 1 mg/body weight or 0.75 mg/g of glucose intraperioneally. Blood glucose was monitored at 0, 15, 30, 60 and 120 minutes after glucose administration.

*Insulin tolerance test*

Mice were fasted for 4-6 hours before injecting 2 mU/g body weight of human recomniant insulin intraperioneally. To avoid stress-induced hyperglycemia, mice were first subjected to a sham injection and blood collection prior to the actual injection of insulin (1). Blood glucose was monitored at 0, 15, 30, 60 and 120 minutes after glucose administration.

*Pyruvate tolerance test*

Mice were fasted for 6 hours before injecting 1.5 mg/g body weight of sodium pyruvate intraperioneally. Blood glucose was monitored at 0, 15, 30, 60 and 120 minutes after glucose administration.

**Supplemental Table 1:** Characteristics of people with diabetic nephropathy and non-diabetic controls

| | Controls (n=11) | Diabetic (n=19) |
|---|---|---|
| Age (years) | 52.1 ± 3.2 | 58.1 ± 2.9 |
| Male gender (%) | 9 (81.8 %) | 10 (52.6 %) |
| Glomerular size ($\mu m^2$) | 12759 ± 2058 | 18664 ± 1738** |
| Diabetic kidney disease stage 1 | N/A | 4 |
| Diabetic kidney disease stage 2 (a and b) | N/A | 8 |
| Diabetic kidney disease stage 3 | N/A | 7 |

Data are means ± SEM or n (%), *p<0.05, Mann-Whitney tests.

**Supplemental Table 2:** Blood glucose, plasma triglycerides and cholesterol over time (14-week study)

| | cASO | | | | LDLR ASO | | | |
|---|---|---|---|---|---|---|---|---|
| | WT | WT APOC3 ASO | OB | OB APOC3 ASO | WT | WT APOC3 ASO | OB | OB APOC3 ASO |
| N | 13 | 14 | 10 | 11 | 19 | 15 | 13 | 13 |
| Sex (Male) | 7 | 8 | 7 | 4 | 8 | 8 | 8 | 5 |
| Baseline | | | | | | | | |
| Body weight (g) | 23.2±1.0 | 22.6±0.8 | 30.5±1.3* | 27.3±1.9* | 30.5±1.3 | 27.3±1.9 | 30.0±1.5* | 28.4±1.4* |
| Blood glucose (mg/dl) | 193.8±13.0 | 180.8±7.1 | 273.5±43.4 | 257.2±36.3 | 181.1± 5.42 | 171.5.2±6.1 | 266.9±23.4 | 319.0±39.4* |
| Cholesterol (mg/dl) | 77.5±2.7 | 75.6±2.9 | 121.4±3.0* | 124.0±6.6* | 121.4±3.0 | 124.0±6.6 | 139.1±8.0* | 110.1±7.7*$^{\#}$ |
| Triglycerides (mg/dl) | 112.7±9.1 | 102.6±8.3 | 133.3±29.8 | 132.4±11.2 | 133.3±29.8 | 132.4±11.2 | 197.4±25.0 | 208.8±37.4 |
| 2 weeks | | | | | | | | |
| Body weight (g) | 29.8±0.9 | 30.2±0.8 | 50.7±1.2* | 47.9±1.3* | 29.2±1.0 | 29.1±1.3 | 44.8±1.0* | 44.0±1.7* |
| Blood glucose (mg/dl) | 173.2±6.3 | 176.9±6.4 | 391.5±45.6* | 330.1±29.4* | 157.3±3.3 | 160.3±4.7 | 428.9±23.3* | 431.5±30.7*$^{\$}$ |
| Cholesterol (mg/dl) | 139.8±9.0 | 130.4±7.7 | 283.7±12.3 | 282.1±16.1 | 428.8±22.1$^{\$}$ | 325.7±29.9$^{\$}$ | 804.7±85.5*$^{\$}$ | 780.8±51.7*$^{\$}$ |
| Triglycerides (mg/dl) | 111.3±10.5 | 54.9±3.2 | 173.1±22.3 | 114.5±11.5 | 165.5±12.3 | 153.7±38.8 | 500.1±53.9*$^{\$}$ | 340.5±39.2*$^{\#\$}$ |
| 4 weeks | | | | | | | | |
| Body weight (g) | 32.6±1.1 | 32.8±1.2 | 61.3±1.1* | 59.4±1.2* | 32.2±0.9 | 31.7±1.3 | 51.3±1.3* | 52.2±1.8* |
| Blood glucose (mg/dl) | 169.5±4.8 | 164.0±4.8 | 299.3±32.8* | 235.3±12.8 | 163.9±5.7 | 166.4±4.8 | 447.6±27.0*$^{\$}$ | 321.7±29.9*$^{\#\$}$ |
| Cholesterol (mg/dl) | 139.8±9.0 | 113.6±6.5 | 293.5±11.3* | 257.1±18.0* | 401.7±13.6$^{\$}$ | 375.7±16.3$^{\$}$ | 829.2±46.8*$^{\$}$ | 971.3±46.0*$^{\#\$}$ |
| Triglycerides (mg/dl) | 89.1±8.2 | 55.9±5.7 | 135.7±12.4* | 109.9±17.9 | 161.8±12.9 | 106.0±10.7 | 467.6±22.2*$^{\$}$ | 407.3±41.7*$^{\$}$ |
| 8 weeks | | | | | | | | |
| Body weight (g) | 36.7±1.3 | 36.1±1.2 | 74.1±1.4* | 69.2±0.9* | 35.9±0.9 | 34.8±1.1 | 55.8±1.4* | 57.9±1.9* |
| Blood glucose (mg/dl) | 161.4±6.1 | 143.2±4.7 | 220.7±24.0 | 194.5±16.9 | 156.5±4.3 | 162.4±6.1 | 372.3±32.6*$^{\$}$ | 203.1±30.5$^{\#}$ |
| Cholesterol (mg/dl) | 135.4±8.1 | 115.0±5.4 | 327±22.32* | 274.6±18.5 | 350.9±13.2$^{\$}$ | 337.5±18.7$^{\$}$ | 946.2±68.8*$^{\$}$ | 798.0±52.0*$^{\#\$}$ |
| Triglycerides (mg/dl) | 84.4±7.2 | 38.3±2.9 | 115.1±15.5 | 64.7±3.1 | 100.5±7.2 | 63.8±3.8 | 473.5±31.4*$^{\$}$ | 263.0±18.7*$^{\#\$}$ |
| 14 weeks | | | | | | | | |
| Body weight (g) | 41.2±1.3 | 39.3±1.0 | 83.7±1.5*$^{\#}$ | 74.7±1.7* | 39.5±1.2 | 38.0±1.1 | 71.6±1.4* | 65.4±2.8* |
| Blood glucose (mg/dl) | 169.5±7.2 | 152.7±4.0 | 242.9±23.8 | 203.1±17.7 | 160.7±4.4 | 159.9±5.1 | 331.8±35.7* | 205.9±32.4$^{\#}$ |
| Cholesterol (mg/dl) | 140.6±7.8 | 116.2±9.1 | 274.2±30.1 | 242.8±23.0 | 319.8±14.2$^{\$}$ | 264.0±19.5$^{\$}$ | 722.6±66.5*$^{\$}$ | 625.8±64.3*$^{\$}$ |
| Triglycerides (mg/dl) | 81.8±12.4 | 27.7±4.1 | 108.5±16.0 | 61.7±5.2 | 93.2±12.0 | 66.6±11.6 | 462.7±31.7*$^{\$}$ | 164.9±18.7$^{\#\$}$ |

Data are means ± SEM, p<0.05 * vs WT (same treatment, e.g. WT vs OB), # vs APOC3 (same treatment), $ vs LDLR ASO within the same treatment.

**Supplemental Table 3:** Urine markers (14-week study)

| | cASO | | | | LDLR ASO | | | |
|---|---|---|---|---|---|---|---|---|
| | WT | WT APOC3 ASO | OB | OB APOC3 ASO | WT | WT APOC3 ASO | OB | OB APOC3 ASO |
| | | | | | | | | |
| Urine creatinine, 8 weeks (mg/ml) | 0.45±0.09 (9) | 0.47±0.03 (13) | 0.19±0.01* (10) | 0.20±0.02* (13) | 0.39±0.05 (10) | 0.37±0.08 (9) | 0.19±0.03 (9) | 0.22±0.02 (13) |
| Urine volume, 8 weeks (µl) | 160±32 | 157±25 | 337±60 | 447±62 | 129±26 | 142±24 | 299±59 | 347±71 |
| Urine albumin, 8 weeks (µg/ml) | 219±110 | 239±23 | 784±103* | 574±69* | 204±50 | 184±44 | 711±68* | 445±88 |
| | | | | | | | | |
| Urine creatinine, 14 weeks (mg/ml) | 0.69 ±0.17 (9) | 0.29±0.05 (11) | 0.29±9.08 (9) | 0.46±0.08 (13) | 0.54±0.19 (15) | 0.29±0.04 (13) | 0.15±0.02 (14) | 0.42±0.1 (12) |
| Urine volume, 14 weeks (µl) | 110±37 | 145±27 | 330±100 | 380±80 | 180±30 | 140±30 | 690±110*# | 150±40 |
| Urine albumin, 14 weeks (µg/ml) | 104±27 | 130±29 | 892±111* | 660±70* | 136±17 | 113±24 | 511±69* | 729±99* |
| Plasma BUN, 14 weeks (mg/dl) | 24.7±1.0 (11) | 21.8±1.4 (13) | 35.0±4.4 (9) | 28.9±9 (13) | 25.5±1.5 (20) | 24.1±1.7 (15) | 43.3±2.5* (13) | 40.2±2.8* (12) |

Data are means ± SEM, $p<0.05$ * vs WT (same treatment), # vs APOC3 (same treatment).

**Supplemental Table 4:** Plasma inflammatory markers (14-week study)

| | cASO | | | | LDLR ASO | | | |
|---|---|---|---|---|---|---|---|---|
| | WT | WT APOC3 ASO | OB | OB APOC3 ASO | WT | WT APOC3 ASO | OB | OB APOC3 ASO |
| N | 7 | 6 | 10 | 13 | 13 | 6 | 13 | 12 |
| 14 weeks | | | | | | | | |
| Plasma IL-18 (pg/ml) | 49.3±18 | 83.7±24.5 | 151.2±49 | 98.5±25 | 161.9±32 | 118.1±53 | 423.4±38*# | 226.9±57 |
| Plasma IL-6 (pg/ml) | 15.0±6.3 | 9.6±3.1 | 15.9±5.2 | 9.2±1.9 | 17.5±4.1 | 16.6±9.8 | 6.8±1.7 | 17.0±5.4 |
| Plasma TNFα (pg/ml) | 8.1±1.0 | 7.7±0.5 | 6.8±0.4 | 6.1±0.3 | 7.6±0.6 | 8.0±1.0 | 7.0±0.4 | 8.3±1.0 |
| Plasma TGFβ1 (pg/ml) | 4256±636 | 3681±469 | 5091±1183 | 5415±1113 | 3688±343 | 2816±515 | 8197±1294* | 8354±1694* |
| Plasma IL-10 (pg/ml) | 25.2±8.6 | 33.7±19.8 | 34.2±7.1 | 41.3±2.9 | 21.6±4.1 | 33.9±3.5 | 52.4±6.6* | 57.4±4.2 |
| Plasma SAA (µg/ml) | 67.0±47 (4) | 83.0±26 (3) | 198.2±74* (4) | 136.5±17 (4) | 43.8±14 (4) | 26.4.0±6 (4) | 115.2±20 (7) | 154.2±17* (10) |

Data are means ± SEM, $p<0.05$ * vs WT (same treatment), # vs APOC3 (same treatment).

**Supplemental Table 5:** Blood glucose, triglycerides, and cholesterol (4-week study)

| | LDLR ASO | | | |
|---|---|---|---|---|
| | WT | WT APOC3 ASO | OB | OB APOC3 ASO |
| N | 11 | 6 | 7 | 8 |
| Sex (Male) | 7 | 4 | 5 | 5 |
| Body weight (g) | 35.2±1.2 | 33.5±0.9 | 47.2±0.6* | 50.1±1.8* |
| Blood glucose (mg/dl) | 158±6 | 159±5 | 536±33*# | 409±33* |
| Cholesterol (mg/dl) | 463±33 | 425±44 | 850±141* | 765±57* |
| Triglycerides (mg/dl) | 143±13 | 94±8 | 385±39*# | 267±29* |
| Monocytes (% of CD45+) | 9.0±0.7 | 10.0±0.9 | 23.8±1.2* | 21.8±1.4* |
| $Ly6C^{hi}$ monocytes (% of CD45+) | 4.0±0.3 | 5.7±0.7 | 12.9±1.1* | 13.1±0.9* |
| $Ly6C^{hi}$ LFA1 (MFI) | 1226±108 | 1361±202 | 1225±81 | 1389±152 |
| $Ly6C^{lo}$ monocytes (% of CD45+) | 4.6±0.5 | 3.9±0.4 | 9.2±1.2* | 7.5±1.3 |
| $Ly6C^{lo}$ CD49D (MFI) | 8010±242 | 8188±397 | 8551±352 | 9003±461 |
| $Ly6C^{lo}$ CX3CR1 (MFI) | 4301±245 | 4050±323 | 3380±311 | 3175±132 |

Data are means ± SEM, p<0.05 * vs WT (same treatment), # vs APOC3 (same treatment).

**Supplemental Table 6:** Blood glucose, triglycerides, and cholesterol (LFA1 study)

| | LDLR ASO | | | |
|---|---|---|---|---|
| | WT Cont Ab | WT LFA1 Ab | OB Cont Ab | OB LFA1 Ab |
| N | 5 | 5 | 5 | 6 |
| Sex (male) | 3 | 4 | 3 | 2 |
| Blood glucose (mg/dl) | 165.0±13.20 | 163.4±8.346 | 525.6±34.67* | 504.7±38.81* |
| Cholesterol (mg/dl) | 354.5±50.75 | 319.2±63.41 | 787.8±25.85* | 743.8±57.20* |
| Triglyceride (mg/dl) | 135.7±15.98 | 123.2±18.11 | 845.2±150.2* | 595.4±70.67* |

Data are means ± SEM, p<0.05 * vs WT (same treatment), # vs LFA1 ab (same treatment).

**Supplemental Table 7:** Blood glucose, triglycerides, and cholesterol (12-week GalNAc APOC3)

| | LDLR ASO | | | |
|---|---|---|---|---|
| | WT cASO | WT APOC3 ASO | OB cASO | OB APOC3 ASO |
| N | 4 | 4 | 4 | 5 |
| Sex (Male) | 4 | 4 | 4 | 5 |
| Baseline | | | | |
| Body weight (g) | 26.7±1.3 | 25.8±0.9 | 31.4.±3.2 | 30.5±1.0 |
| Blood glucose (mg/dl) | 152.0±8.4 | 158.5±8.5 | 370.8±72* | 313±41* |
| 4 weeks | | | | |
| Body weight (g) | 34.0±0.4 | 33.8±0.9 | 41.8.±1.2* | 40.1±1.8* |
| Blood glucose (mg/dl) | 156.8±6 | 156.8±14 | 480.3±36* | 353.2±31* |
| Cholesterol (mg/dl) | 206.8±20 | 176.2±26 | 606.2±13*# | 434.0±51* |
| Triglyceride (mg/dl) | 121.3±16 | 60.0±10 | 232.4±22*# | 107.8±21 |
| 8 weeks | | | | |
| Body weight (g) | 33.5±2.6 | 36.6±1.4 | 45.9.±1.9* | 48.1±1.0* |
| Blood glucose (mg/dl) | 153.8±8 | 158.3±12 | 478.5±63* | 398.6±60* |
| Cholesterol (mg/dl) | 145. 4±11 | 115.0±21 | 532.0±52*# | 297.9±68* |
| Triglyceride (mg/dl) | 88.0±11 | 43.9±8 | 339.2±47*# | 93.9±26 |
| | | | | |
| 12 weeks | | | | |
| Body weight (g) | 36.9±0.5 | 37.8±1.4 | 52.0.±1.1* | 53.3±2.0* |
| Blood glucose (mg/dl) | 125.3±10 | 137.0±3.0 | 425.5±58*# | 254.8±41 |
| Cholesterol (mg/dl) | 159.8±13 | 158.5± 26 | 685.2±63* | 523.4±90* |
| Triglyceride (mg/dl) | 56.4±7.6 | 33.9±3.0 | 252.1±14*# | 100.2±21* |

Data are means ± SEM, p<0.05 * vs WT (same treatment), # vs APOC3 (same treatment).

**Supplemental Table 8:** Antibodies and key reagents

| Antibody/reagent | Clone/product ID | Manufacturer | Dilution/Dose |
|---|---|---|---|
| CD45-FITC | 30-F11 | eBioscience | 1:500 (0.1 μg/100 μl) |
| CD115-APC | AFS98 | eBioscience | 1:100 (0.2 μg/100 μl) |
| GR1-PE-Cy7 | RB6-8C5 | eBioscience | 1:1000 (0.05μg/100 μl) |
| CD49D-PE (α4 integrin) | PS/2 | Southern Biotech | 1:1000 (0.01 μg/100 μl) |
| CD11B-PE | M1/70 | eBioscience | 1:500 (0.1 μg/100 μl) |
| F4/80-PE-Cy7 | BM8 | eBioscience | 1:200 (0.2 μg/100 μl) |
| Viability dye e450 | 65-0863-14 | eBioscience | 1:1000 |
| Mac-2 | CL8942AP | Cedarlane | 1 μg/ml |
| Fluoresbrite yellow green microspheres (latex particles) | 1715210 | Polysciences, Inc. | 250 μl/mouse (diluted 1:4) |
| Alpha Smooth muscle actin | Ab5694 | Abcam | 0.2 μg/ml |
| APOB (biotinylated)<br>Control (BAF108) | BAF3556 | RnD systems | 1:50 |
| Tyramide-AlexaFluor488 (100x stock) used for APOB staining | B40953 | ThermoFisher | 1x |
| APOE | AB183597 | Abcam | 1:2000 |
| Perilipin 2 | NB110-40877 | Novus | 1:400 |
| ICAM1 | 14-0542-82 | eBioscience | 1:100 |
| biotinylated-*Lycopersicon esculentum-derived* lectin | B-1175 | Vector Laboratories | 0.1 mg/mouse |
| EasySep Biotin Positive selection kit II | 17683 | Stem cell technologies | NA |
| APOC3 ELISA | Ab217777 | Abcam | 1:250,000 (plasma) |
| Albumin ELISA | 1011 | Ethos Bioscience | 1:50 (urine) |
| The Creatinine Companion (Creatinine Assay) | 1012 | Ethos Bioscience | 1:20 (urine) |
| IL-18 ELISA | 88-50618 | Invitrogen | 1:2 (plasma) |
| IL-6 Mouse ProQuantum Immunoassay Kit | A43656 | Invitrogen | 1:5 (plasma) |
| TNF alpha Mouse ProQuantum Immunoassay Kit | A43658 | Invitrogen | 1:10 (plasma) |
| TGF-β1 ELISA | BMS608-4 | Invitrogen | 1:20 (plasma) |
| IL-10 ELISA | 88-7105-22 | Invitrogen | 1:2 (plasma) |
| ICAM1 blocking ab | clone M17/4; (BE0006) | BioXcell | 300 μg/mouse x3 times per week |
| Control ab (against trinitrophenol, not expressed in mammals) | clone 2A3 (BE0089) | BioXcell | 300 μg/mouse x3 times per week |
| Mouse Insulin | clone K36AC10 (I2018) | Sigma Aldrich | 1:20,000 |
| APOC3 ASO (Gen 2) | CCAGCTTTATTAGGGACAGC | Ionis | 50 mg/kg/week |
| Control for APOC3 ASO (Gen 2) | CCTTCCCTGAAGGTTCCTCC | Ionis | 50 mg/kg/week |
| LDLR ASO | CTTTATCTTTAACCTC | Ionis | 5 mg/kg/week |
| Control for LDLR ASO | GGCCAATACGCCGTCA | Ionis | 5 mg/kg/week |
| APOC3 GalNAc | CCAGCTTTATTAGGGACAGC | Ionis | 10 mg/kg/week |
| GalNAc control | CCTTCCCTGAAGGTTCCTCC | Ionis | 10 mg/kg/week |

## Supplemental table 9: Primer sequences

| Gene | Forward primer | Reverse primer |
|---|---|---|
| *Rn18s* | CATTAAATCAGTTATGGTTCCTTTGG | CCCGTCGGCATGTATTAGCT |
| *Ccl2* | TTAAAAACCTGGATCGGAACCAA | GCATTAGCTTCAGATTTACGGGT |
| *Il1b* | GGGCTGCTTCCAAACCTTTG | TGATACTGCCTGCCTGAAGCTC |
| *Tnfa* | CCTGTAGCCCACGTCGTAG | GGGAGTAGACAAGGTACAACCC |
| *Il6* | TAGTCCTTCCTACCCCAATTTCC | TTGGTCCTTAGCCACTCCTTC |
| *Icam1* | GGCATTGTTCTCTAATGTCTCC | GCTCCAGGTATATCCGAGCTTC |
| *Vcam1* | TGCACAGTCCCTAATGTGTATCC | GACTTTATGCCCATTTCCTCCA |
| *Apoc3* | TACAGGGCTACATGGAACAAGC | CAGGGATCTGAAGTGATTGTCC |
| *Perilipin 2* | CTTGTGTCCTCCGCTTATGTC | GCAGAGGTCACGGTCTTCAC |

## Supplemental references

1. Hull RL, Hackney DJ, Giering EL, and Zraika S. Acclimation Prior to an Intraperitoneal Insulin Tolerance Test to Mitigate Stress-Induced Hyperglycemia in Conscious Mice. *J Vis Exp.* 2020(159).

Supplemental figure 1

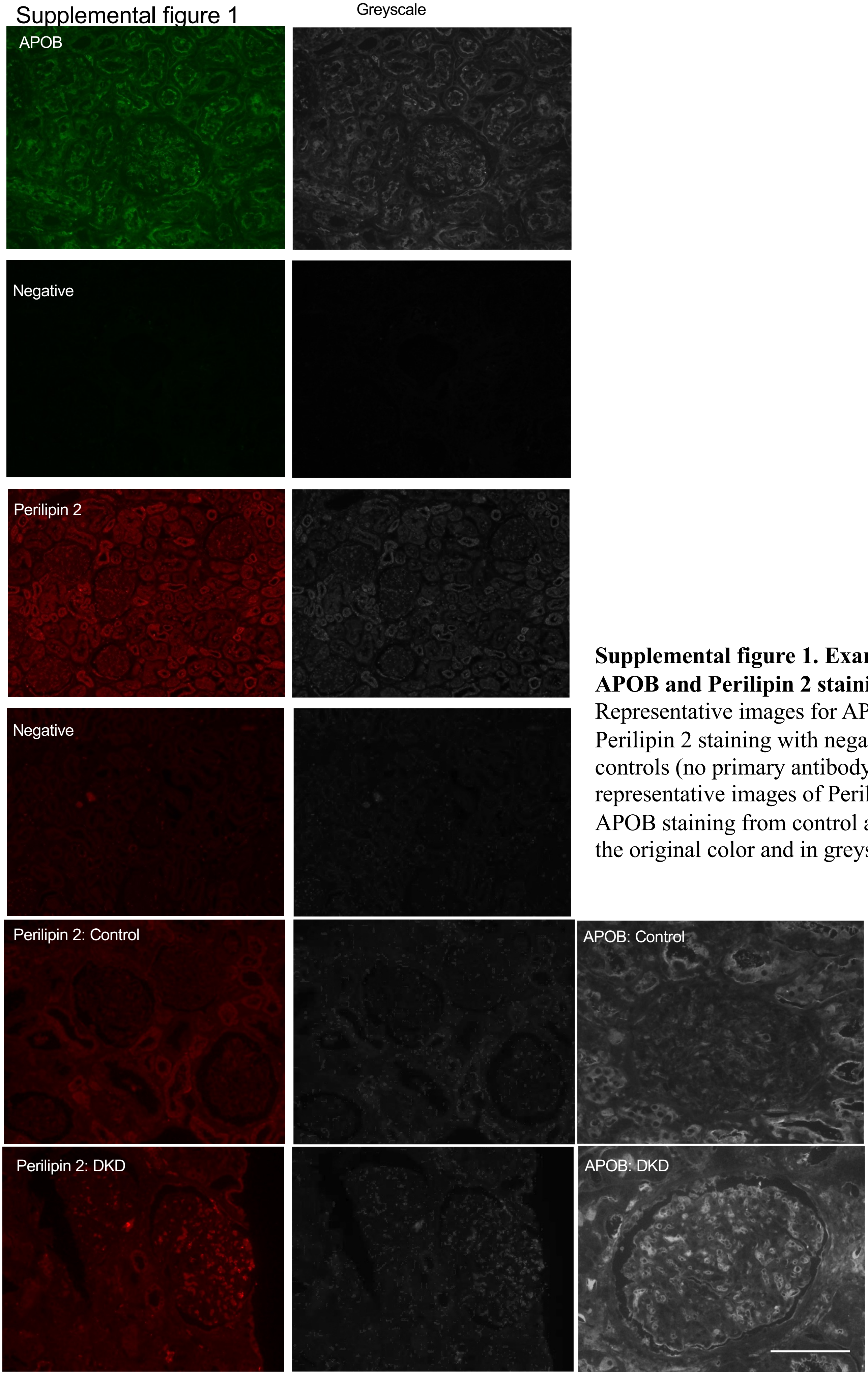


**Supplemental figure 1. Examples of APOB and Perilipin 2 staining**. Representative images for APOB and Perilipin 2 staining with negative controls (no primary antibody) and representative images of Perilipin 2 and APOB staining from control and DKD in the original color and in greyscale.

Supplemental figure 2

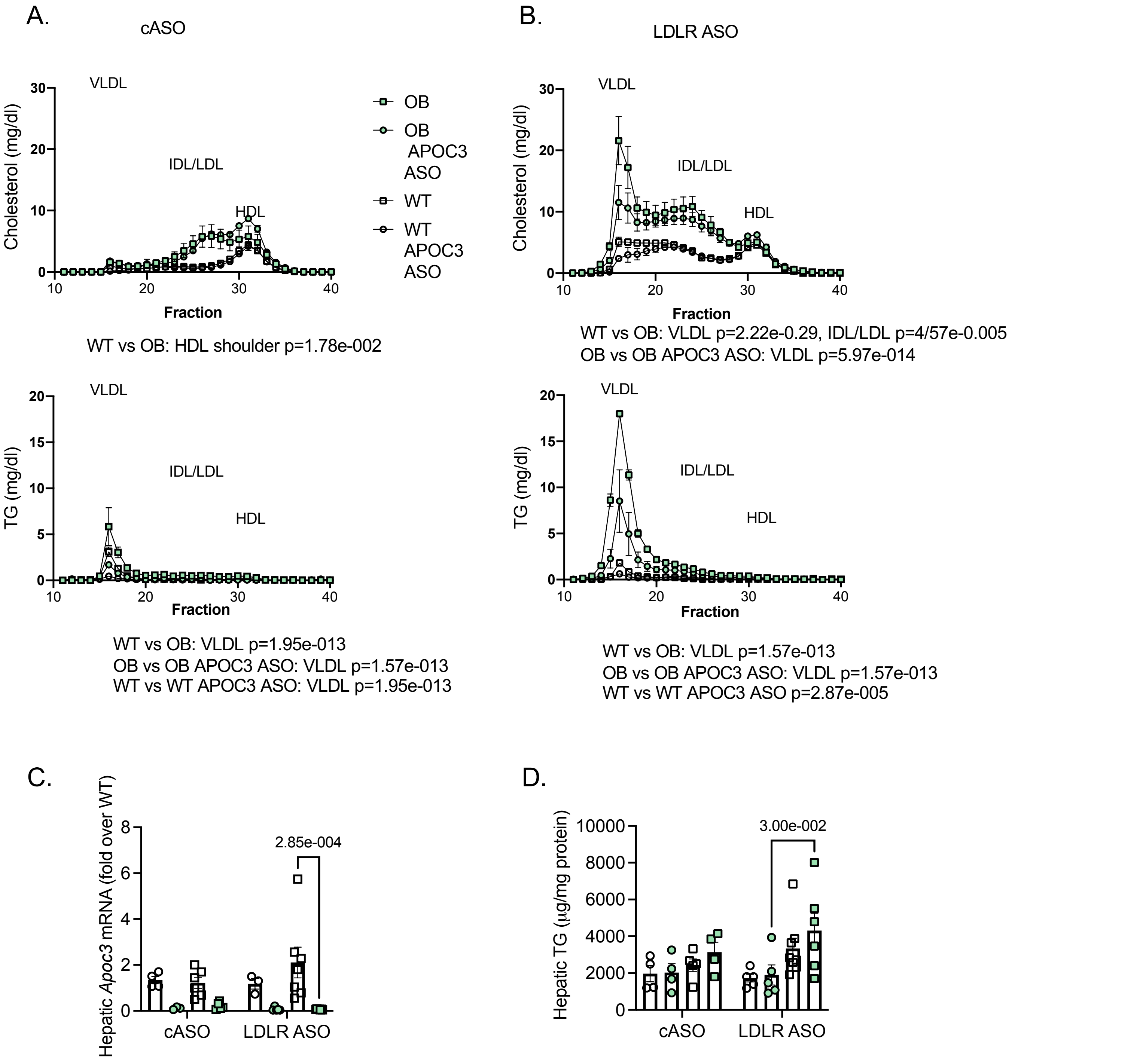


**Supplemental figure 2. APOC3 ASO treatment reduces VLDL**. Briefly, WT and leptin-deficient OB mice were treated with either a control antisense oligonucleotide (cASO) or LDLR ASO. Within each group, a subset was treated with either a cASO or an ASO to APOC3. Mice were then placed on a high-fat diet for 14 weeks. FPLC separation of plasma and analysis of cholesterol and triglycerides (TG) in cASO-treated mice (A) and LDLR ASO treated mice (B) (N=4). C. Hepatic expression of *Apoc3* mRNA (N=3-7). D. Hepatic triglycerides (TG; N=4-8). Data expressed as mean ± SEM. Data was analyzed by 2-WAY ANOVA followed by Tukey's multiple comparisons test. Text under the graph indicates the overall significance.

Supplemental figure 3

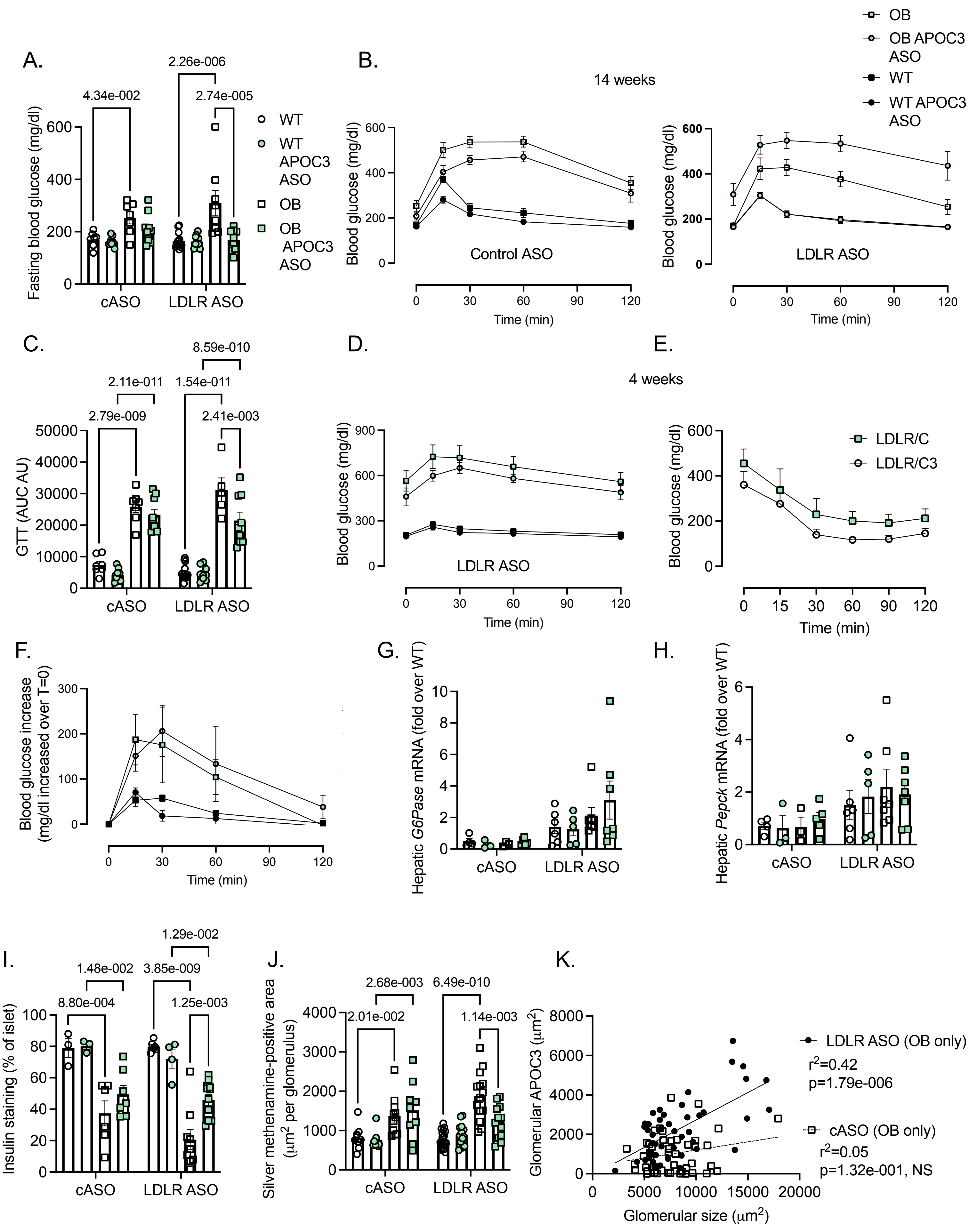

**Supplemental Figure 3. APOC3 ASO treatment results in preservation of islet insulin**. Mice were treated as in Figure S2. A. Fasting glucose. B. Intraperiontal (IP) glucose tolerance test (GTT) with 1 mg glucose/g body weight. C. Area under each individual mouse's curve from the GTT. D-F. GTT, insulin tolerance test (ITT) and pyruvate tolerance test (PTT) in mice treated for 4 weeks with LDLR ASO and APOC3 ASO or cASO (4-week study). D. IP-GTT with 0.75 mg/g. E. IP-ITT using 2mU of insulin/g of body weight F. Pyruvate tolerance test with 1.5 mg pyruvate/g body weight. Data expressed increment in blood glucose over time point 0. G. Hepatic mRNA expression of *G6pase* from the 14-week study (N=3-7). H. Hepatic mRNA expression of *Pepck* from the 14-week study (N=3-7). I. Pancreas insulin staining from the 14-week study (N=3-12). J. Glomerular silver methenamine stain in the 14-week study (N=8-21). K. Correlation between APOC3 and glomerular size on a per glomerulus size in OB mice only (n=48-92, from 5-7 mice). Data expressed as mean ± SEM. Data was analyzed by 2-WAY ANOVA followed by Tukey's multiple comparisons test. Text under the graph indicates the overall significance. N as indicated in Figure 5A unless otherwise noted.

Supplemental figure 4

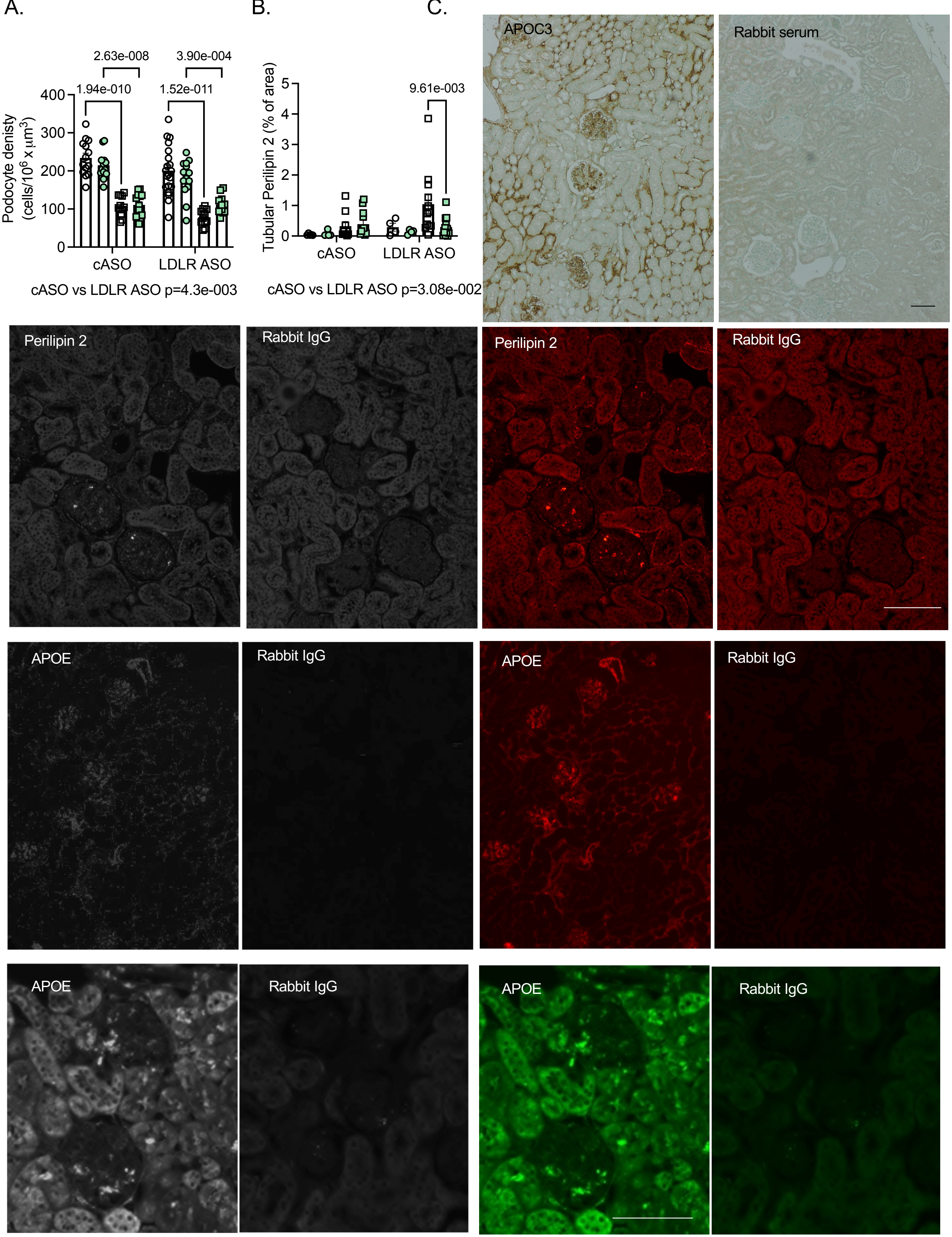

**Supplemental figure 4. Examples of APOC3, Perilipin 2 staining, APOE and APOB**. A. Glomerular WT-1 (podocytes) staining in the 14-week study (N=11-20). B. Quantification of non-glomerular perilipin 2 staining in 14-week study (N=4-12). C. Representative images for APOC3, Perilipin 2 staining, APOE and APOB with negative controls (corresponding IgG control) in the original color and in greyscale. Data expressed as mean ± SEM. Data was analyzed by 2-WAY ANOVA followed by Tukey's multiple comparisons test. 2 statistical outlier were removed from the perilipin 2 analysis.

Supplemental figure 5

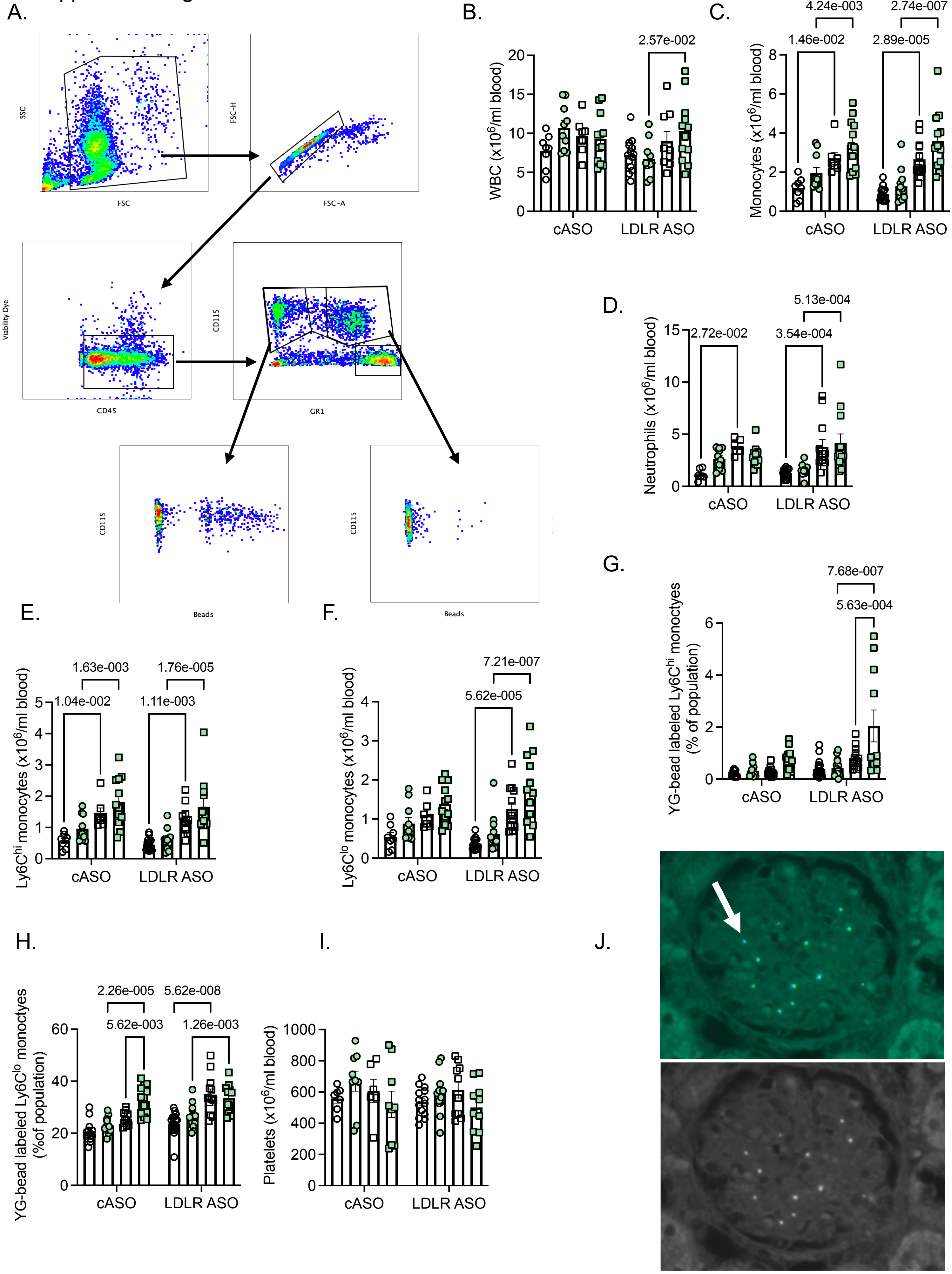

**Supplemental figure 5. Flow cytometry of circulating leukocytes**. Mice were treated as in Supplemental figure 2. A. Representative gating strategy to identify bead-positive monocyte populations in blood. B. White blood cells (WBC) from whole blood measured using a hemavet. C-H, cell populations based on flow cytometry combined with hemavet analysis. C. Blood monocytes. D. Blood neutrophils. E. Blood Ly6C$^{hi}$ monocytes. F. Blood Ly6C$^{lo}$ monocytes. G. Yellow-green (YG) positive blood Ly6C$^{hi}$ monocytes. H. YG-positive blood Ly6C$^{lo}$ monocytes. I. Platelets, based on hematvet analysis. J. Representative image of YG beads in glomerulus. Data expressed as mean ± SEM. Data was analyzed by 2-WAY ANOVA followed by Tukey's multiple comparisons test. N as indicated in Figure 2D.

Supplemental figure 6

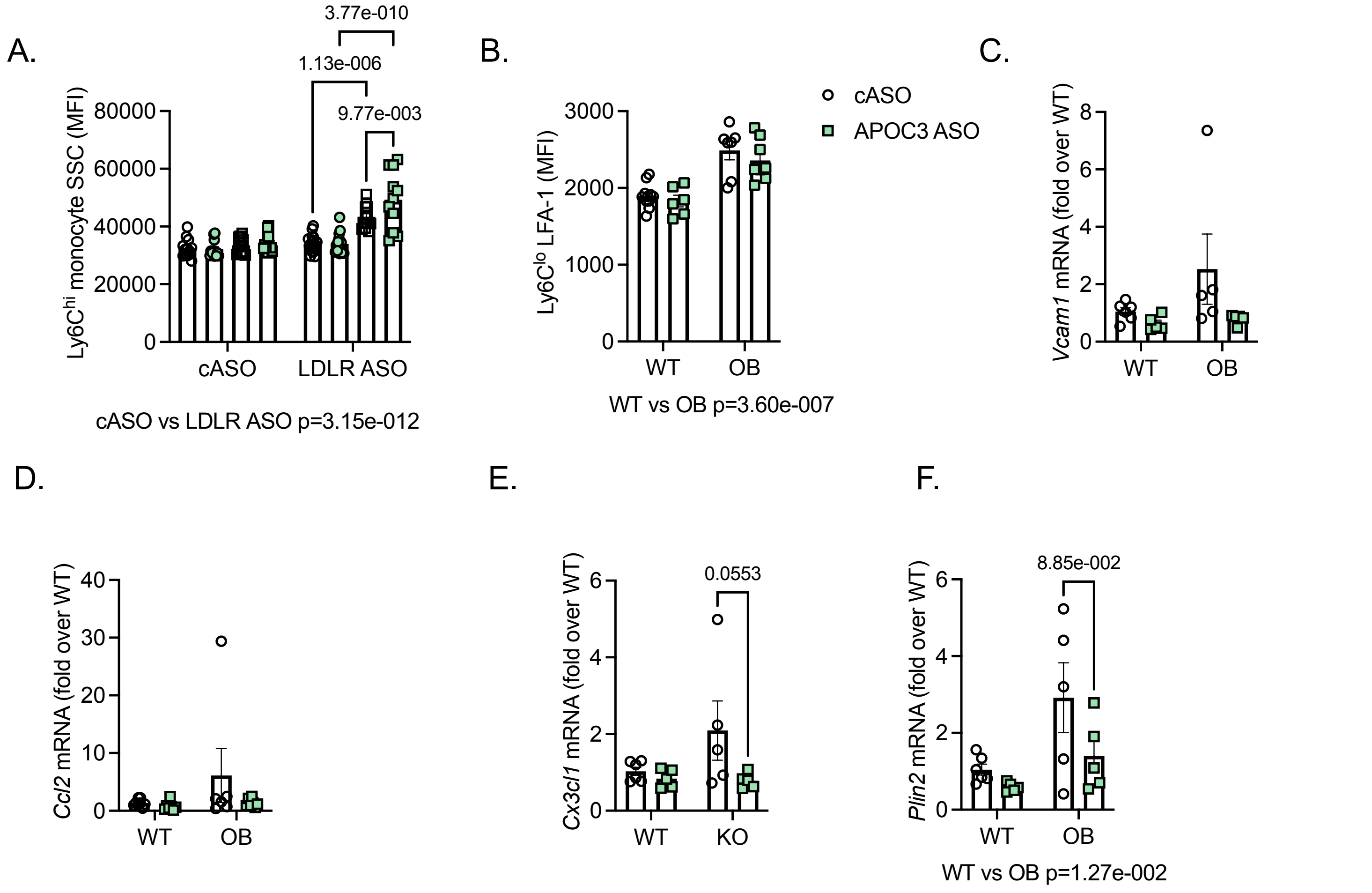


**Supplemental figure 6. Leptin-deficiency increases Ly6C$^{lo}$ monocyte LFA1**. A. Side scatter (SSC) in Ly6C$^{hi}$ monocytes, which is indicative of lipid loading from the mice in the 14-week study. B. LFA1 expression in Ly6C$^{lo}$ monocytes from BTBR WT and OB mice were treated for 4 weeks with LDLR ASO and APOC3 ASO or cASO (4-week study). C. *Vcam1* mRNA in isolated kidney cortex endothelial cells. D. *Ccl2* mRNA in isolated kidney cortex endothelial cells. E. *Cx3Ccr1* mRNA in isolated kidney cortex endothelial cells. F. *Plin2* mRNA in isolated kidney cortex endothelial cells. Data expressed as mean ± SEM. Data was analyzed by 2-WAY ANOVA followed by Tukey's multiple comparisons test. N as indicated in Figure 5A.

Supplemental figure 7

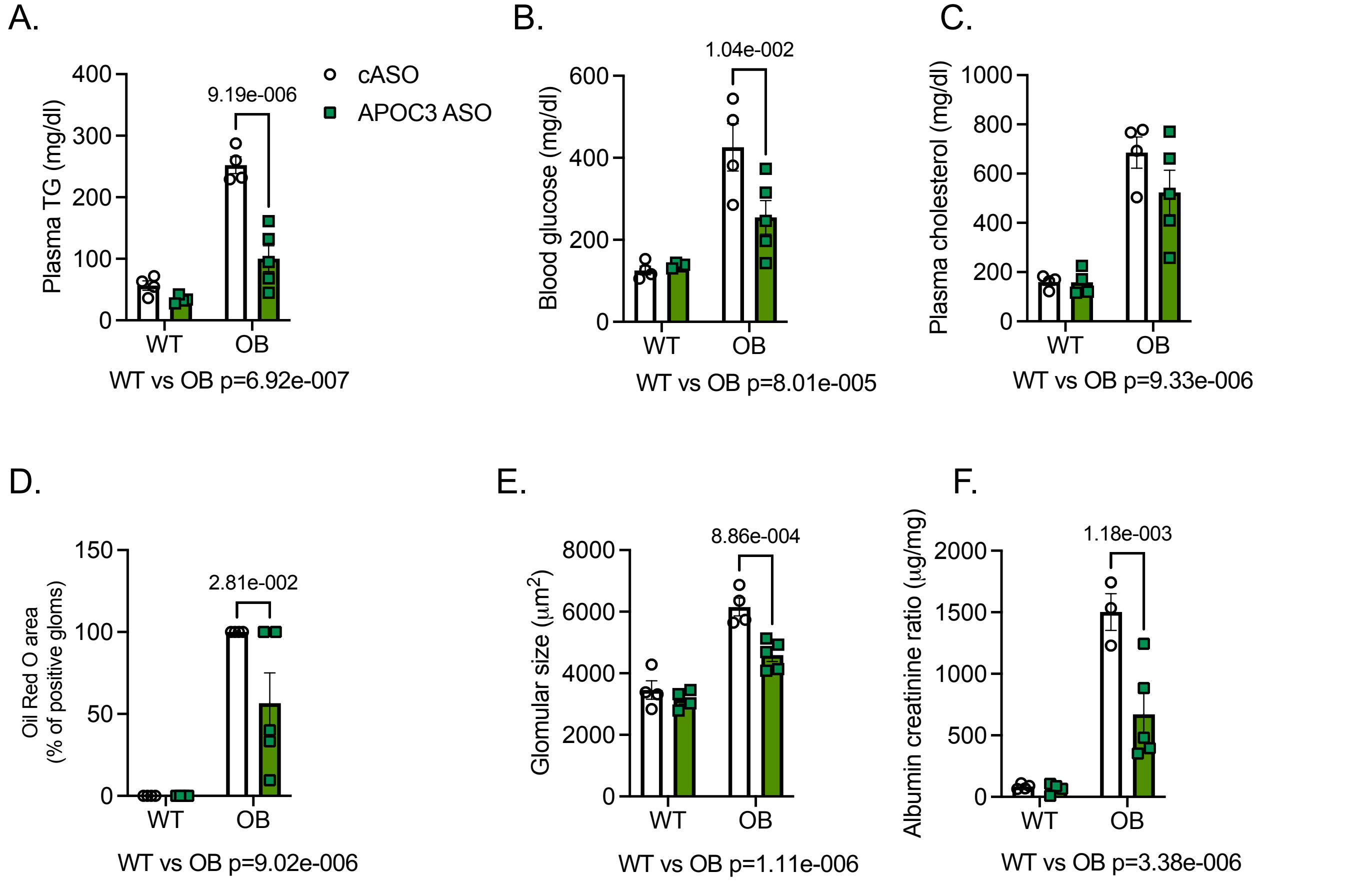


**Supplemental figure 7. APOC3 silencing using GalNAc-modified APOC3 ASO**. BTBR WT and OB mice were treated for 12 weeks with LDLR ASO and APOC3 ASO or cASO while maintained on a low-fat diet. A. Plasma triglycerides (TG) at 12 weeks. B. *Ad lib* fed blood glucose at 12 weeks. C. Plasma cholesterol at 12 weeks. For additional time points, see **Supplemental Table 8**. D. Glomerular oil red o-staining expressed as the overall % of positive glomeruli (independent of extent of staining within a positive glomerulus). E. Glomerular size. F. Urine albumin to creatinine ratio at 12 weeks. Data expressed as mean ± SEM. Data was analyzed by 2-WAY ANOVA followed by Tukey's multiple comparisons test. N=4-5.

Supplemental figure 8

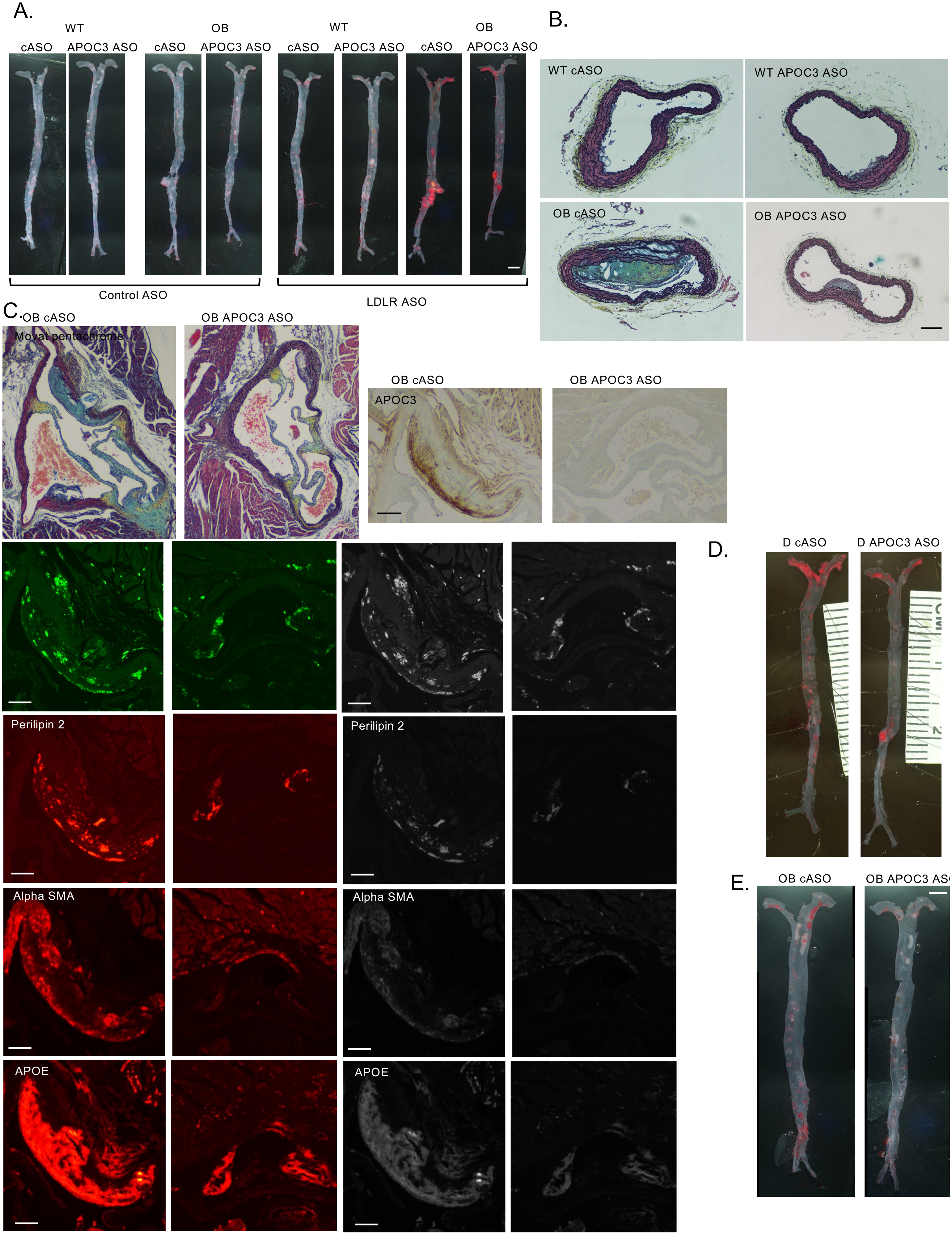

**Supplemental figure 8. APOC3 silencing reduces atherosclerosis**. Representative images of *en face* aortic lesions as assessed by Sudan IV staining from the 14-week study. B. Representative images of Movat pentachrome stained brachiocephalic artery lesions from the 14-week study. C. Representative images of Movat pentachrome stained aortic sinus lesions, APOC3, Mac-2, Perilipin 2, alpha-smooth muscle actin (SMA) and APOE from OB (LDLR ASO) treated with cASO and APOC3 ASO (14-week study) in the original color and in greyscale. D. Representative images of *en face* aortic lesions as assessed by Sudan IV staining from the type 1 diabetes study. Representative images of *en face* aortic lesions as assessed by Sudan IV staining from the 12-week study using the GalNAc-ASO. Scale bar in A and E is 2 mm, 100 μm in B and C.
